# The genetic basis of salinity tolerance traits in Arctic charr (*Salvelinus alpinus*)

**DOI:** 10.1186/1471-2156-12-81

**Published:** 2011-09-21

**Authors:** Joseph D Norman, Roy G Danzmann, Brian Glebe, Moira M Ferguson

**Affiliations:** 1 Department of Integrative Biology, University of Guelph, Guelph, Ontario, N1G 2W1, Canada; 2 Department of Fisheries and Oceans, St. Andrews Biological Station, St. Andrews, New Brunswick, E5B 2L9, Canada

**Keywords:** Arctic charr, salmonid fishes, salinity tolerance, Na^+^/K^+^-ATPase, osmoregulation, whole-genome duplications; homeologies, duplicated genes

## Abstract

**Background:**

The capacity to maintain internal ion homeostasis amidst changing conditions is particularly important for teleost fishes whose reproductive cycle is dependent upon movement from freshwater to seawater. Although the physiology of seawater osmoregulation in mitochondria-rich cells of fish gill epithelium is well understood, less is known about the underlying causes of inter- and intraspecific variation in salinity tolerance. We used a genome-scan approach in Arctic charr (*Salvelinus alpinus*) to map quantitative trait loci (QTL) correlated with variation in four salinity tolerance performance traits and six body size traits. Comparative genomics approaches allowed us to infer whether allelic variation at candidate gene loci (e.g., *ATP1α1b, NKCC1, CFTR*, and *cldn10e*) could have underlain observed variation.

**Results:**

Combined parental analyses yielded genome-wide significant QTL on linkage groups 8, 14 and 20 for salinity tolerance performance traits, and on 1, 19, 20 and 28 for body size traits. Several QTL exhibited chromosome-wide significance. Among the salinity tolerance performance QTL, trait co-localizations occurred on chromosomes 1, 4, 7, 18 and 20, while the greatest experimental variation was explained by QTL on chromosomes 20 (19.9%), 19 (14.2%), 4 (14.1%) and 12 (13.1%). Several QTL localized to linkage groups exhibiting homeologous affinities, and multiple QTL mapped to regions homologous with the positions of candidate gene loci in other teleosts. There was no gene × environment interaction among body size QTL and ambient salinity.

**Conclusions:**

Variation in salinity tolerance capacity can be mapped to a subset of Arctic charr genomic regions that significantly influence performance in a seawater environment. The detection of QTL on linkage group 12 was consistent with the hypothesis that variation in salinity tolerance may be affected by allelic variation at the *ATP1α1b *locus. *IGF2 *may also affect salinity tolerance capacity as suggested by a genome-wide QTL on linkage group 19. The detection of salinity tolerance QTL in homeologous regions suggests that candidate loci duplicated from the salmonid-specific whole-genome duplication may have retained their function on both sets of homeologous chromosomes. Homologous affinities suggest that loci affecting salinity tolerance in Arctic charr may coincide with QTL for smoltification and salinity tolerance traits in rainbow trout. The effects of body size QTL appear to be independent of changes in ambient salinity.

## Background

The life history of anadromous salmonids entails migration between freshwater and seawater environments. To ensure that internal ion concentrations remain homeostatic in the face of abrupt changes in ambient salinity, an individual's osmoregulatory mechanisms must switch between states of ion absorption (i.e., hyper-osmoregulation) and ion excretion (i.e., hypo-osmoregulation). These changes are particularly important in salmonids whose reproductive cycle is dependent upon anadromous behaviour. As described by the seawater mitochondria-rich cell model, hypo-osmoregulation is primarily achieved by mechanisms associated with mitochondria-rich cells in gill tissue [1]. In conjunction with pavement cells and accessory cells, mitochondria-rich cells form the epithelial layer of gill tissue, where the removal of Cl^- ^is facilitated by three interdependent membrane-bound ion transporters. An electrochemical gradient across the basolateral membrane is actively maintained by Na^+^/K^+^-ATPase pumps that exchange intracellular Na^+ ^for extracellular K^+^, thereby driving Na^+^/K^+^/2Cl^- ^(NKCC) cotransporters to move Cl^- ^from blood plasma into the cell. Once the intracellular electrochemical equilibrium is reached, Cl^- ^exits the cell through cystic fibrosis transmembrane conductance-like regulator anion channels (CFTR) embedded in the apical membrane. Na^+ ^secretion is thought to occur passively through leaky cation-selective paracellular pores between accessory cells and mitochondria-rich cells [1,2]. A current hypothesis suggests that alternate claudin isoforms may confer differential permeability characteristics at these junctions [3,4], as evident in kidney tissue [5].

Research at the molecular level has largely focused on Na^+^/K^+^-ATPase genes, where two isoforms in particular have important implications for hyper- and hypo-osmoregulation [6,7]. Seawater immersion is correlated with transcriptional up-regulation of *ATP1α1b*, and down-regulation of *ATP1α1a*, whereas the reciprocal pattern is evident in freshwater. This phenomenon has been observed in rainbow trout (*Oncorhynchus mykiss*) [6,7], Atlantic salmon (*Salmo salar*) [7-9], Arctic charr (*Salvelinus alpinus*) [7], and brown trout (*Salmo trutta*) [10].

Variation in salinity tolerance is well documented within and among the Salmoninae (*Oncorhynchus, Salmo *and *Salvelinus*) [7,8,11-14]. *Salvelinus *species are typically far less anadromous than *Salmo *species [15] and are considered to be less efficient osmoregulators [7,16]. The evidence that variation in salinity tolerance capacity of salmonids is attributed in part to underlying genetic variation is mostly indirect. For example, different strains of Arctic charr show wide variation in seawater-induced mortality, where the salinity tolerance capacity of some individuals is commensurate with that of Atlantic salmon [17]. Other experiments with salmonids conclude that the basis for such variation likely has a genetic component [18-20]. The most convincing evidence comes from a study on rainbow trout, where variation in genomic regions was associated with seawater-induced fluctuations in Na^+ ^and Cl^- ^ion concentrations in blood plasma [21].

The rich evolutionary history of salmonids has provided multiple opportunities for the pseudogenization, neofunctionalization and subfunctionalization of genes involved in osmoregulation. The 1-2-4 model of vertebrate evolution demonstrates that the vertebrate ancestor underwent two genome duplication events (i.e., 1R, 2R) prior to the divergence of gnathostomes [22]. Support for a third, fish-specific genome duplication event (3R; the 1-2-4-8 model) is evident in the striking pattern of doubly conserved synteny blocks among the genomes of *Tetraodon nigroviridis *and *Homo sapiens *[23]. It is now generally accepted that a fourth, salmonid-specific genome duplication (4R), in the form of an autotetraploidization event, occurred in the salmonid ancestor some 25-100 million years ago [24]. Although disomic inheritance has been largely restored in modern salmonids, segregation patterns characteristic of tetrasomic inheritance and the occurrence of multivalent formations during meiosis continue to be observed [24,25]. Multiple Na^+^/K^+^-ATPase genes have been detected and mapped within the genomes of Atlantic salmon and rainbow trout, where some duplicated loci reside on homeologous linkage groups [26,27]. Although unmapped, transcription patterns of *ATP1α1a *and *ATP1α1b *isoforms in gill tissue provide evidence that they are conserved in Arctic charr [7]. Furthermore, comparative analysis among homologous chromosome blocks within the salmonids can facilitate a more direct comparison of the Na^+^/K^+^-ATPase genes with model species such as rainbow trout [28,29].

A complete physiological model inclusive of all the relevant mechanisms that affect variation in salinity tolerance capacity would be ideal prior to characterizing their underlying genetic bases. Nonetheless, the mitochondria-rich cell model remains useful, since genome scans coupled with quantitative trait locus (QTL) analyses can be utilized to identify chromosomal regions correlated with variation in salinity tolerance performance traits. QTL analyses are necessary as the traits by which seawater performance is assessed can not be objectively divided into discrete groups, for their distributions are typically continuous, and as such are not amenable to study using classical Mendelian genetics. Once QTL are identified, comparative genomics methods can determine if they occupy regions sharing homology with chromosomes holding salinity tolerance candidate genes (e.g., *ATP1α1a, NKCC1, CFTR, cldn10e*) in the sequenced genomes of 3R teleosts, such as zebrafish (*Danio rerio*) and medaka (*Oryzias latipes*). Confirmation of homology would advance hypotheses that variation in salinity tolerance capacity is a function of allelic variation at those loci.

In this study we employ a genome-scan approach to identify the genomic regions in Arctic char that correlate with variation in four salinity tolerance performance traits: Na^+^/K^+^-ATPase activity, blood plasma osmolality, and specific growth rates from two distinct time intervals. We describe multiple QTL for each trait among several linkage groups, and find that some QTL co-localize with the putative locations of candidate genes predicted by comparative genomics with zebrafish and medaka. We also describe putative homologous QTL from comparisons with smoltification and salinity tolerance QTL locations in the rainbow trout genome [21,30].

## Results

### Genetic Maps

Four sex-specific genetic maps were generated from the parents of two full-sib families (i.e., 10, 12; see Additional files 1, 2, 3, 4). Each map was comprised of over 100 markers arranged into a minimum of 27 linkage groups. Unlinked markers represented three to four linkage groups per map. Linkage group names were ascribed following designations from existing Arctic charr maps [28,31]. A total of 35 linkage groups from a potential 39 in Arctic charr were represented by at least one informative marker among all families.

### QTL analysis

#### Salinity tolerance traits

**Figure 1:**
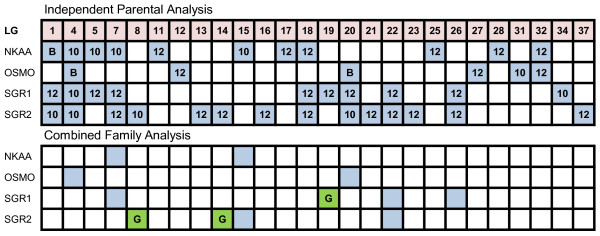
**Linkage group (LG) distribution of genome-wide (green) and chromosome-wide (blue) salinity tolerance QTL in the Arctic charr (*Salvelinus alpinus*) genome**. NKAA Na^+^/K^+^-ATPase activity; OSMO blood plasma osmolality; SGR1 specific growth rate from June 12 to August 28, 2008; SGR2 August 29 to November 14, 2008; B QTL detected in both families; G genome-wide significant QTL. The analyses used to establish QTL significance are noted in Additional files 5, 6, and 7.

QTL with chromosome-wide significance were identified on multiple linkage groups for each seawater performance trait. QTL for Na^+^/K^+^-ATPase activity, blood plasma osmolality, specific growth rate 1, and 2, each localized to 11, 6, 10, and 14 linkage groups, respectively, across both families (Figure 1). Co-localization of QTL among traits was evident on 9 linkage groups (i.e., AC-1, -4, -5, -7, -18, -20, -22, -26, and -32). Most notably were AC-1, -4, -7, -18, and -20, which were associated with QTL for three or more traits. QTL for Na^+^/K^+^-ATPase activity explained between 5.1 and 9.2% of experimental variation (see Additional file 5), while the PEV for blood plasma osmolality was highest for QTL on AC-4 (14.1%), -12 (13.1%), and -20 (19.9%). QTL on AC-19 and -22 explained the most variation in specific growth rate 1, at 14.2 and 10.3%, respectively, while the greatest variation in specific growth rate 2 was associated with AC-1 (10.5%), and AC-21 (11.8%) (see Additional file 6). When all four parents were combined in one analysis, 11 QTL were detected on 8 linkage groups among all four traits (i.e., AC-4, -7, -8, -14, -15, -19, -22, -26) (see Additional file 7). Co-localization of QTL for different traits was evident on AC-7, -15, and -22. The combined analysis also identified three QTL with genome-wide significance, which were restricted to specific growth rate 1 (i.e, AC-19) and 2 (i.e., AC-8, -14). Several QTL were identified on multiple linkage groups exhibiting homeologous affinities (see Additional file 8).

#### Body weight and condition factor

**Figure 2:**
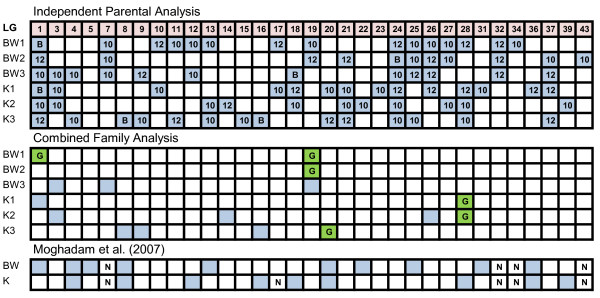
**Linkage group (LG) distribution of genome-wide (green) and chromosome-wide (blue) body weight and condition factor QTL in Arctic charr (*Salvelinus alpinus*) and a comparison with Moghadam et al. (2007)**. BW1 body weight in June 2008; BW2 body weight in August; BW3 body weight in November 2008; K1 condition factor in June 2008; K2; condition factor in August 2008; K3 condition factor in November 2008; B QTL detected in both families; G genome-wide significant QTL. N not included in the analysis by Moghadam et al. (2007). The analyses used to establish QTL significance are noted in Additional files 9 and 10.

Analyses were conducted on body weight and condition factor data collected from fish exposed to freshwater (body weight 1, condition factor 1) and seawater (body weight 2 and 3, condition factor 2 and 3). Across both families, chromosome-wide significant QTL for body weight 1, 2, and 3 each localized to 16, 12, and 12 linkage groups, respectively, while respective QTL for condition factor 1, 2, and 3 were identified on 15, 11, and 14 linkage groups (Figure 2). QTL for all body weight and condition factor traits co-localized to AC-1 and AC-24. The most variation in body weight 1 was explained by QTL on AC-27 (10.1%) and AC-32 (12.9%) (see Additional file 9). A QTL for body weight 2, associated with the same marker interval as body weight 1 on AC-27 (i.e., CA383830 - Sal9UoG), accounted for 17.2% of variation, while a QTL on AC-7 explained 10.6% of variation in body weight 3. Loci explaining the most variation in condition factor 1 and 2 were identified on AC-1 (condition factor 1, 10.6%), -28 (condition factor 1, 12.6%), and -21 (condition factor 2, 11.5%). Combined family analysis identified 16 chromosome-wide significant QTL on 11 linkage groups among all body weight and condition factor trait categories (see Additional file 10). Six genome-wide significant QTL were identified on 4 linkage groups (i.e., AC-1, -19, -20, -28). QTL for body weight 1 and 2 co-localized to AC-19, whereas QTL for condition factor 1 and 2 co-localized to AC-28.

## Discussion

### Salinity Tolerance QTL

We found several QTL for seawater traits (i.e., Na^+^/K^+^-ATPase activity, blood plasma osmolality, specific growth rate 1, and 2) over 26 linkage groups. Among these, genome-wide significant QTL were restricted to AC-8, -14 (specific growth rate 2) and -19 (specific growth rate 1). Co-localization of QTL for multiple traits occurred on nine linkage groups (i.e., AC-1, -4, -5, -7, -18, -20, -22, -26, and 32). We also detected several QTL on linkage groups exhibiting homeologous affinities [28,29] (see Additional file 8) suggesting the possibility that salinity tolerance may be affected by functional gene duplicates derived from the 4R genome duplication.

**Figure 3:**
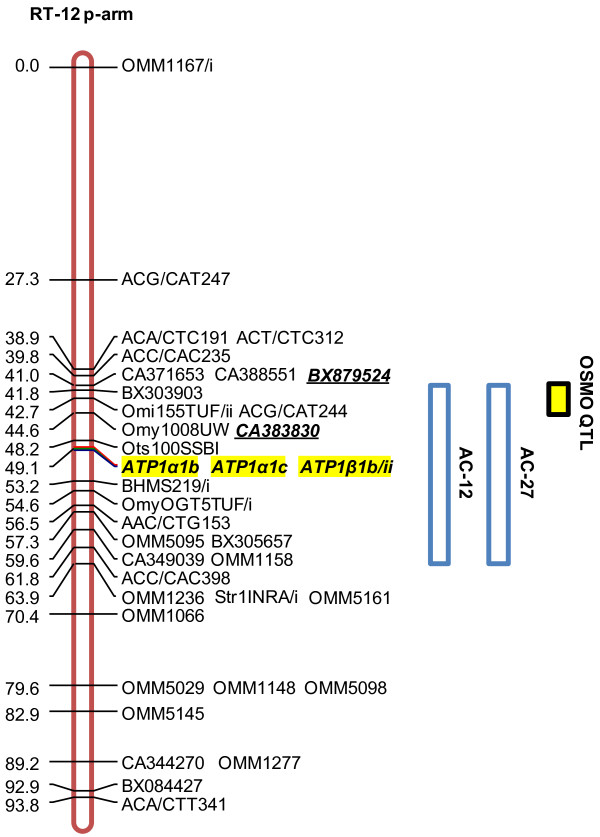
**Rainbow trout (*Oncorhynchus mykiss*) linkage group RT-12 (p-arm) (red) and homologous regions (blue) in Arctic charr (*Salvelinus alpinus*) containing QTL for blood plasma osmolality (yellow)**. QTL- linked markers are bold and italicized; candidate genes highlighted yellow; OSMO blood plasma osmolality; ATP1 sodium-potassium ATPase enzyme; α1b, α1c, and β1b are isoform designations; rainbow trout linkage group and homologies with Arctic charr obtained from Danzmann et al. (2005). Some markers have been removed for clarity.

Comparative analyses with species where potential salinity tolerance candidate genes are mapped suggest that some of these regions are homologous with salinity tolerance QTL. In the case of *ATP1α1b *and *NKCC1*, direct comparisons can be made with the rainbow trout and Atlantic salmon genomes. For unmapped genes, such as *CFTR *and claudins, rainbow trout can be used as a proxy for comparisons with the sequenced genomes of zebrafish and medaka [32]. Based on the location of *ATP1α1b *in rainbow trout and Atlantic salmon [26,27] we predicted that QTL for salinity tolerance traits would localize to AC-12 and -27. We found that QTL for blood plasma osmolality did indeed localize to each of these linkage groups (see Figure 3 and Additional file 5), which supports the hypothesis that variation in salinity tolerance capacity may be influenced by allelic variation at *ATP1α1b *loci.

The QTL effect on AC-12 may also be associated with variation in claudins. It has been suggested that permeability characteristics of Na^+ ^ions through paracellular junctions between mitochondria-rich cells and accessory cells in gill tissue may be correlated with claudin isoform identity [3,4]. In fact, among the claudin 26 isoforms identified in Atlantic salmon, qPCR analysis shows that mRNA of *cldn10e *is significantly elevated after seawater exposure [4]. Interspecific homologies suggest that the genomic positions of *ATP1α1b *and claudin isoform 10e (*cldn10e*) may have converged to a single linkage group in salmonids despite the fact that their apparent homologues are located on separate linkage groups in both zebrafish (Zv9 database; *ATP1α1b *on Dr-9, GenBank:NP_571765; *cldn10e *on Dr-6, GenBank:XM_678711) and medaka (MEDAKA1 database; *ATP1α1 *on Ol-2, ENSEMBL:ENSORLG00000002047; *cldn10 *on Ol-21, ENSEMBL:ENSORLG00000017717). Further, Dr-6 and Ol-2 show extensive synteny (supported by ≥ 3 markers) with RT-12 and AS-22 where the Na^+^/K^+^-ATPase α-isoform clusters are located [26,27], which suggests that *cldn10e *and *ATP1α1b *may reside on the same linkage group in salmonids. Chromosomes Dr-6/9 and Ol-2/21 are also derived from the C ancestral chromosome grouping in teleost fishes, and this ancestral lineage is the most conserved syntenic block on the rainbow trout linkage groups RT-12p/16p and Atlantic salmon AS-22qb (homologous with AC-12/27) chromosome blocks [32]. This further supports a likely conserved evolutionary origin for the *ATP1α1b *and *cldn10e *genes.

Genomic homologies among Arctic charr linkage groups containing seawater QTL with the genomes of zebrafish and Atlantic salmon suggest that the QTL on AC-4 may stem from the combined effects of multiple candidate loci. Two isoforms of *NKCC1 *(a and b) are known to exist [8], one of which has been mapped to AS-17 in Atlantic salmon (unpublished data) [8]. Among the Arctic charr linkage group that share homology with AS-17 (i.e, AC-4, -9, -21, and -28) [28], AC-4 (i.e., Na^+^/K^+^-ATPase activity, specific growth rate 1 and 2), -21 (i.e., specific growth rate 2) and -28 (i.e., Na^+^/K^+^-ATPase activity) contain salinity tolerance QTL. Considering the role of *NKCC1 *in ion excretion, putative effects related to allelic variation at this locus would more likely be reflected by variation in Na^+^/K^+^-ATPase activity levels rather than growth measurements. Thus if variation at *NKCC1 *affected salinity tolerance capacity in the present study, it may not be unexpected to find QTL on AC-4 and/or -28 arising from allelic variation at this locus. Presently, a single *NKCC1 *locus is annotated in the zebrafish genome, whereas two *NKCC1 *loci have been characterized in European eel (*Anguilla anguilla*) [33]. This suggests that the tetraploid salmonid ancestor might have possessed up to four functional *NKCC1 *genes. As such it is possible that the Arctic charr genome could contain multiple functionally active *NKCC1 *isoforms, which could be associated with QTL on AC-4 and -28. This is also supported by the fact that a putative homeologous affinity has been detected between AC-4/28 [29].

Comparative genomics suggests that a *CFTR *locus may also reside on AC-4. BLASTN searches of the *CFTR *gene in zebrafish (ENSEMBL v.59; http://www.ensembl.org/) identified significant homology with complete cDNA sequences of *CFTR-I *(GenBank:AF155237) and *CFTR-II *(GenBank:AF161070) from Atlantic salmon [34]. Both copies coalesced to the same region on Dr-18 (*CFTR-I*, E-value = 5.1E^-95^; *CFTR-II*, E-value = 1.2E^-106^; Zv9; ENSEMBL:ENSDARG00000041107), supporting the annotation of only a single *CFTR *gene in zebrafish. Similarly, in medaka and stickleback single gene copies for *CFTR *have been localized to an unassigned scaffold (E-value = 0) and to chromosome Ga-XIX at position 10.186Mb (E-value = 0), respectively. This also suggests that duplicates of *CFTR *in salmonids may be derived from the 4R whole-genome duplication. The known locations of the *CFTR *gene in zebrafish and stickleback suggest an origin of this gene from the K ancestral lineage of teleost fishes which may thus share homology to linkage groups RT-7, -15, and 27q and possibly more extensive regions on RT-6p [32]. Linkage group RT-27q shares some homology with AC-19a and/or AC-19b and is syntenic with AC-4a [28,29], suggesting that these linkage groups may house *CFTR *duplicates. Incongruence of trait-specific QTL among AC-4 (i.e., Na^+^/K^+^-ATPase activity, blood plasma osmolality, specific growth rate 1and 2) and -19 (i.e, specific growth rate 1) suggests that the QTL on AC-4 confers a stronger effect on mitochondria-rich cell physiology than that of AC-19, indicating that AC-4 is a better candidate for the location of *CFTR*. However, it is also possible that each of AC-4 and -19 may contain a *CFTR *isoform, for as mentioned previously, two *CFTR *genes have been detected in the Atlantic salmon genome [34]. Moreover, over a small region AC-4 and -19 appear to share a homeologous affinity [29]. As postulated for *ATP1α1b *and *cldn10e*, copies of *NKCC1 *and *CFTR *may have converged to the same linkage group in salmonids (e.g., AC-4 in Arctic charr), or retained expression of one of the duplicate copies of these genes (i.e, on AC-4), if derived from AC-4/19 duplicates, despite being on separate linkage groups in zebrafish (i.e., Dr-8 and -18).

**Figure 4:**
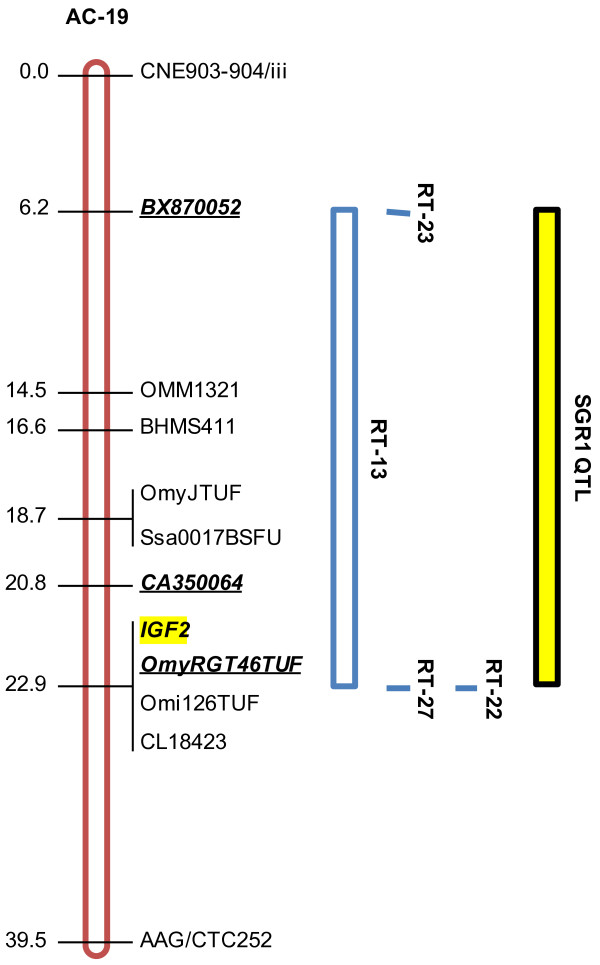
**Seawater growth QTL (yellow) on Arctic charr (*Salvelinus alpinus*) linkage group AC-19 (red) and homologous regions (blue) in rainbow trout (*Oncorhynchus mykiss*)**. QTL- linked markers are bold and italicized; candidate genes highlighted yellow; SGR1 specific growth rate June 12 to August 28, 2008; *IGF2 *insulin-like growth factor 2; Arctic charr linkage group and homologies with rainbow trout obtained from Danzmann et al. (2005).

Our inference that genetic variation at *IGF2 *could contribute to the variation in salinity tolerance suggests that candidate genes not identified in the current mitochondria-rich cell model are relevant to ion regulation in fish gill tissue. Comparison with a reference map for Arctic charr [28,29] revealed that the genome-wide significant QTL for specific growth rate 1 on AC-19 (BX870052/i, *P *= 0.001) was proximal to an *IGF2 *locus (Figure 4; Table 1). Furthermore, QTL across all seawater performance traits were located on AC-4, to which a second *IGF2 *locus has been mapped [35]. Given that *IGF2 *mRNA levels in rainbow trout appear to be growth hormone-dependent [36], *IGF2 *expression in gill tissue may be the product of direct regulation by growth hormone, which could have acute effects on hypo-osmoregulation that are independent of somatic growth [37,38]. For instance, in brown trout (*Salmo trutta*), growth hormone has been connected to changes in the size and number of mitochondria-rich cells, and by extension, with the concentration of Na^+^/K^+^-ATPase and Na^+^/K^+^/2Cl^- ^cotransporters [39]. Further, in rainbow trout Le Bras *et al*. [21] describe QTL for gill weight on linkage groups RT-15 and -27, which contain *IGF1 *and *IGF2 *loci, respectively [35]. The association of a QTL for specific growth in seawater (i.e., specific growth rate 1) on AC-19 may be the product of a dual action of growth hormone, whereby the stimulation of somatic growth (directly by growth hormone or indirectly by *IGF1*) is concurrent with gill tissue growth and mitochondria-rich cell propagation initiated by growth hormone-induced activation of *IGF2*. Interestingly, similar to the putative syntenic co-localizations predicted for the Na^+^/K^+^-ATPase and *cldn10e *genes, the predicted locations for *NKCC1 *(i.e., AC-4 and -28) and *CFTR *(i.e., AC-4 and -19) isoforms in Arctic charr overlap with the confirmed locations of *IGF2 *(i.e, AC-4 and -19) [35], suggesting the possibility salinity tolerance genes may cluster throughout the genome.

**Table 1:** Comparison of salinity tolerance and body size trait QTL (*P *≤ 0.05) in Arctic charr (*Salvelinus alpinus*) with growth-related genes mapped by Moghadam et al. (2007)

LG	Gene	Salinity Tolerance QTL	Body Size QTL
3	*MYF5, IGF1/i*	None detected	BW3, K1, K2
4	*IGF2/i*	NKAA, OSMO, SGR1, SGR2	BW3, K3
14	*PACAP*	SGR2	K2
19	*IGF2/i*	SGR1	BW1, BW2, BW3
20	*GH2*	OSMO, SGR1, SGR2	K1, K3
24	*IGF1/ii*	None detected	BW1, BW2, BW3, K1, K2, K3
27	*PACAP*	OSMO	BW1, BW2, K2

LG linkage group; *MYF5 *myogenic factor 5; *IGF1 *insulin-like growth factor 1; *IGF2 *insulin-like growth factor 2; *PACAP *pituitary adenylate cyclase-activating polypeptide; *GH2 *growth hormone 2; NKAA Na^+^/K^+^-ATPase activity; OSMO blood plasma osmolality; SGR1 specific growth rate from June 12, 2008 to August 28, 2008; SGR2 specific growth rate from August 29, 2008 to November 14, 2008; BW1 body weight on June 12, 2008; BW2 body weight on August 28, 2008; BW3 body weight on November 14, 2008; K1 condition factor on June 12, 2008; K2 condition factor on August 28, 2008; K3 condition factor on November 14, 2008.

Allelic diversity at *IGF2 *loci may also contribute to variation in salinity tolerance capacity in non-salmonids. Transcriptional responses in multiple teleost species demonstrate that mRNA production levels of several genes not included in the mitochondria-rich cell model are contingent upon seawater exposure [40-43]. *IGF2 *has been implicated in salinity tolerance capacity in black-chinned tilapia (*Sarotherodon melanotheron*), where it was reported that gill tissue *IGF2 *mRNA levels were elevated in response to seawater [44].

Some of the salinity tolerance QTL reported here may co-localize to homologous linkage groups containing smoltification QTL in rainbow trout [30]. This is evident for the RT-16p and AC-12/27 [28,29] linkage groups, which share homologous affinities. The genetic marker associated with variation in skin reflectance (i.e., Omy77DU) on RT-16 [30] maps to within 0.8 cM of the EST linked to a QTL for blood plasma osmolality on AC-27 (i.e., CA345149) [32]. These QTL may derive from a single locus and thereby be pleiotropic, or alternatively, may be part of a salinity tolerance or smoltification-related gene-cluster. Intriguingly, thyroid hormone levels have been associated both with purine deposition (i.e., skin reflectance) and changes in Na^+^/K^+^-ATPase activity [15,38], suggesting that, irrespective of genomic position, these loci may be regulated by thyroid hormone.

Certain salinity tolerance QTL shared tentative homologies with smoltification QTL in rainbow trout. The rainbow trout reference map reveals that a major QTL associated with multiple smoltification-related traits was linked to markers on RT-20q (i.e., OMM5108, OMM5017) [30,32], which has homologous affinities with AC-13b (a putative metacentric) and AC-34 [28,29]. AC-13 contains a QTL for specific growth rate 2 (i.e., OMM1174-OMM5180) that spans both the a- and b-arms while a QTL for specific growth rate 1 has been localized (i.e., OMM1657-OMM5312) on AC-34.

Other genome-wide significant QTL showed little to no homology with smoltification QTL in rainbow trout. Although the marker linked to a specific growth rate 2 QTL on AC-8b (i.e., OmyRGT6TUF) is found on RT-17 (centromeric regions) and RT-22p [32], where QTL for reflectance (i.e, OMM1117) and body shape (i.e., Ssa289) were localized [30], the respective markers are each > 30 cM away from OmyRGT6TUF [32], indicating that these QTL are likely not homologous. Also, a QTL on AC-14 was not homologous with any smoltification QTL in rainbow trout. These discrepancies could be related to differences in experimental design, for Nichols *et al*. [30] exposed their fish only to freshwater. This suggests that the effects conferred by the aforementioned QTL may be specifically induced by seawater exposure, and furthermore, that the genes involved in preparing a fish for migration to seawater (i.e., smoltification) are not necessarily the same as those involved in hypo-osmoregulation. Differences could also be due to species, ontological status, environmental conditions, or any combination thereof.

For the salinity tolerance QTL in rainbow trout described by Le Bras *et al*. [21], putative QTL homologies were apparent with the chromosome-wide and genome-wide QTL detected from the combined analysis in Arctic charr within the following regions: RT-4 (AC-4); RT-10 (AC-15); RT-25 (AC-4); and RT-26 (AC-4, -25) for physiological traits. Homologies with QTL for gill weight on RT-9q (AC-20), RT-9p (AC-23) and RT-26 (AC-4, -25 and possibly -22) were also evident. Also, the strong QTL region detected on AC-19 may share homology to the QTL regions on RT-19 although the Arctic charr markers appear to lie more in the central cluster of RT-19 linkage group, while those reported span the RT-19q arm [21]. Similarly a homeologous region to AC-19 (i.e., AC-32) overlaps the QTL region reported on RT-23q [21]. Multiple homology assignments for AC-8 exist within the rainbow trout genome (i.e., RT-2p; RT-3q; RT-7q and RT-10q) with the largest tract homologous to RT-3q. Nonetheless, homologies to the region reported on RT-10 [21] cannot be excluded at present. Finally, AC-14 shares homology with RT-24q and also shares marker synteny with two locations around the centromere on RT-19 and the p-arm. This region may be related to the QTL localized to RT-19 [21].

### Body Size QTL

We identified genome-wide significant body size QTL on AC-1 (body weight 1), -19 (body weight 1 and 2), -20 (condition factor 3), and -28 (condition factor 1 and 2). A marker linked to a QTL for body weight 1 on AC-1 (i.e., BX311884) was previously associated with body weight QTL in Arctic charr [45]. Further, the association of AC-1 with QTL for Na^+^/K^+^-ATPase activity, specific growth rate 1 and 2 is suggestive of its relevance to salinity tolerance. The close proximity of QTL for body weight 1 (BX311884) and Na^+^/K^+^-ATPase activity (OMM1330) suggests they may be controlled by a pleiotropic locus, for their respective markers are separated by only 2.1 cM [28,29]. However, at this time alternative explanations such as tight linkage among loci cannot be ruled out, since each marker interval will likely contain clusters of several genes [46].

Our genome-wide significant results for condition factor support the findings of Moghadam *et al*. [45], who described a locus on AC-28 that affected this trait. In fact, the same genetic marker that we found to be associated with the QTL for condition factors 1 and 2 on AC-28, was identified by Moghadam *et al*. [45] (i.e., Omi34TUF) as localizing the QTL. We also identified a QTL for body weight 1 on AC-28, however according to reference maps [28,29] the associated marker (i.e., OMM1459) is 49 cM away from Omi34TUF, suggesting that separate QTL regions on this linkage group affect either trait. These regions are clearly autonomous in rainbow trout, for OMM1459 (RT-23, p-arm) and Omi34TUF (RT-1) are located on distinct linkage groups [32].

QTL detected within the first sampling time period remained constant temporally, indicating that there were no gene × time interaction effects. Further, the QTL effects appeared to be largely unaffected by changes in ambient salinity, given that some QTL were associated with both freshwater (body weight 1) and seawater (body weight 2 and 3) traits, suggesting that gene × environment interaction effects were also minimal. In fact, this pattern was evident among several linkage groups affiliated with body size QTL (i.e., AC-1, -7, -19, -24, -25, -26, -27). This result is somewhat unexpected given that gene × environment interactions have been observed for growth and gene expression in salmonid fishes [47].

Although uncommon, the effects of some QTL were restricted to either seawater or freshwater. Though tentative observations were made, the discernment of which loci, if any, that could have exerted such effects was difficult given that our experiment was not designed to make such assessments. The only QTL with putative effects specific to seawater exposure was located on AC-9 (body weight 3 and condition factor 3). A QTL on AC-4 also appeared to be seawater-specific (i.e., QTL for only body weight 3 and condition factor 3). However, AC-4 has also been associated with body weight QTL in freshwater Arctic charr [45], and thus separate loci on AC-4 may influence growth differentially in either rearing environment. Identifying QTL with potential freshwater -specific effects proved more difficult, for only a single freshwater sample was collected. Regardless, our data tentatively suggest that freshwater QTL reside on AC-10 and -17, both of which contain QTL for body weight 1 and condition factor 1. Moghadam *et al*. [45] did not find QTL on either linkage group, despite using freshwater Arctic charr in their study.

We were able to confirm several QTL previously detected by other researchers. In our evaluation of body weight and condition factor of individuals in freshwater, we found three body weight 1 QTL (i.e., AC-1, -13, and -25), and four condition factor QTL (i.e., AC-18, -20, -28, -36) that coincided with those of Moghadam *et al*. [45]. Approximately 60% of the growth-related QTL detected by Moghadam *et al*. [45] were also detected in the current study (see Figure 2). This agreement is not surprising, for both studies used the same Arctic charr strain (i.e., Fraser River) and performed genome scans using genetic markers derived from the same reference mapping panel. Though the fish in the present study were 1.5 years old, those used by Moghadam *et al*. [45] were only 1 year-old, which could explain some of the discrepancies, as QTL regions are differentially expressed as salmonids age [48].

This study was the first to use a genome-scan approach to assess the genetic basis of salinity tolerance in Arctic charr. However, it involved a relatively low density genome scan which may encompass 10's to 100's of differentially interacting genes spanning the existing interval regions mapped. Under these constraints, it is perhaps more likely to observe little if any overlap in reported QTL positions rather than the relative congruence that has been reported in QTL studies to date. There is a need for multiple replicated studies examining the association between putative QTL regions and trait expression before a final consensus can be reached as to which regions tend to have the strongest influences upon any given trait. While family based and age-related differences are likely to exist, major strain-specific differences may also exist [10,14], highlighting the need for additional studies of this important physiological trait.

## Conclusions

We identified several genomic regions associated with seawater performance traits. The detection of QTL on AC-12 provided further support for the hypothesis that genetic variation at the *ATP1α1b *locus may confer an effect on salinity tolerance. In addition, the detection of strong QTL on AC-19 led us to propose that genetic variation at the *IGF2 *locus may also affect hypo-osmoregulation. Multiple instances of apparent conserved effects among homeologous linkage groups within Arctic charr were observed, and some salinity tolerance QTL appeared to be in regions homologous with salinity tolerance and smoltification QTL in rainbow trout [21,30]. Body size QTL were not affected by changes in ambient salinity, and coincidence with the findings of others further confirmed multiple freshwater -based body size QTL in Arctic charr. Though these findings are only suggestive, they provide support for the justification of future work in elucidating the genetic basis of salinity tolerance, for it is clear that several regions in the Arctic charr genome affect the salinity tolerance capacity of the individual, thereby providing a foundation for more detailed candidate gene-based experiments.

## Methods

### Strain Background and Rearing

Six families (denoted 9, 10, 11, 12, 18, and 28) were produced in November, 2006, at the Coastal Zones Research Institute (Shippigan, New Brunswick, Canada) using full-sib crosses from an F3 generation originally derived from an anadromous Arctic charr population from the Fraser River, Labrador, Canada. In the summer of 2007, approximately 150 progeny from each family were PIT (passive integrated transponder) tagged and transferred to St. Andrews Biological Station (St. Andrews, New Brunswick, Canada) where they were reared in two cylindrical 1 m^3 ^freshwater tanks, under controlled simulated-natural photoperiod and water temperature regimes. Tissue was collected from the adipose fin from all individuals for genetic analysis in March, 2008. On June 12, 2008, body weight and fork length measurements were obtained for all individuals (= body weight 1), that were then randomly sorted among six cylindrical 1 m^3 ^tanks to facilitate growth and as a prelude to future experiments. The tanks were equally stocked (~120 fish·tank^-1^) and families equally represented (~20 fish·family^-1^tank^-1^). Each tank was supplied with filtered, aerated freshwater (9.9-10.7°C, flow rate 18 L·min^-1^, dissolved O_2 _10.0-10.6 mg·L^-1^), and covered with a clear Plexiglas lid. To minimize the confounding effects associated with a naturally changing photoperiod, a 16 h-light/8 h-dark photoperiod regime was maintained for the duration of the sampling period (June 9, 2008 to July 6, 2008), after which a simulated-natural photoperiod was restored. Light was provided by 30 W incandescent bulbs, centred approximately 0.75 m above each tank. Feeding occurred daily to satiation with Skretting Optiline salmonid feed pellets (Skretting, Bayside, NB, Canada). Fish were held in these conditions for 7-14 days prior to seawater introduction, dependent upon the tank sampling order.

### Experimental Protocol

All tanks were converted to seawater in the period of June 19, 2008 to June 26, 2008, at a rate of one tank·day^-1^. For each tank, freshwater was replaced with filtered seawater over a 24-hour period: at 6-h intervals freshwater and seawater inputs were proportionately decreased and increased, respectively, such that after 24-h the tank consisted of 100% seawater (31-33‰, 10.5-11.9°C, flow rate 18 L·min^-1^, dissolved O_2 _8.1-11.4 mg·L^-1^). Fish were fasted for 24-h prior to sampling. All sampling was preceded by anaesthetization with tricaine methanesulfonate (MS 222; 150 mg·L^-1^), and concluded with replacement of the individual into a seawater recovery tank. Ninety-six hours post-full seawater exposure, blood was collected by caudal puncture with a heparinized syringe (500 U mL^-1 ^heparin) and placed on ice. Within 15 minutes of sample acquisition, blood was centrifuged at 13500 g for 4 minutes at 4°C, after which plasma was immediately removed and frozen in liquid nitrogen at -80°C for future analysis. Ten days post full seawater exposure non-lethal gill biopsies [49] were collected from the same individuals. Upon excision, tissue samples were immersed in 500 μL of ice-cold SEI buffer (250 mM sucrose, 10 mM EDTA, 50 mM imidazole, pH 7.3) in 2 mL cryovials and were frozen within 0.5 h in liquid nitrogen at -80°C for future analysis. We sampled ten days post-seawater exposure as historically this is when differences in Na^+^/K^+^-ATPase activity in Arctic charr have been observed [7]. Individuals remained in seawater from June 19-26, 2008, to November 14, 2008. Body weight and fork length measurements were taken again on August 28, 2008 and November 14, 2008, to facilitate the calculation of specific growth rates. At each sampling time, fish were monitored for signs of early maturity, as indicated by secondary sexual characteristics (darkening body colour, kype development) or gamete extrusion. Mortality was monitored daily.

### Phenotypic measurements

Blood plasma osmolality (mOsmol·kg^-1^) was measured using a vapour pressure osmometer (Wescor model 5520; Wescor Inc., Utah, USA). Na^+^/K^+^-ATPase activity (μmol ADP·mg protein^-1^·h^-1^) was determined spectrophotometrically following the methods of McCormick [49]. Gill filaments were homogenized on ice in SEI buffer for 30 s using a disposable pestle grinder system (Fisher Scientific). Homogenates were centrifuged at 5000 g for 30 s at 4°C to separate insoluble material from the supernatant, which was used directly in the assay mixture (189 mM NaCl, 42 mM KCl, 10.5 mM MgCl_2_, 50 mM imidazole, 0.7 mM ATP, 2.8 mM phospho(enol)pyruvate, 0.22 mM NADH, 4.0 U mL^-1 ^lactic dehydrogenase, 5.0 U mL^-1 ^pyruvate kinase, pH 7.5) or the assay mixture plus ouabain (0.7 mM), a Na^+^/K^+^-ATPase enzyme inhibitor. Na^+^/K^+^-ATPase activity was measured in triplicate at 340 nm for 10 minutes using a SpectraMax 190 microplate reader (Molecular Devices, Sunnyvale, CA, USA) maintained at 25°C. Protein concentration was determined with a commercial bicinchoninic acid (BCA) protein assay kit (Pierce, Rockford, Illinois, USA). Specific growth rates were calculated for two periods: June 12 to August 28, 2008 (= Specific Growth Rate 1), and August 29, to November 14, 2008 (= Specific Growth Rate 2), with the formula, G = [Ln(W_t_) - Ln(W_i_)]/t, where W_t _was weight at time t, and W_i _was the initial weight [50]. Fulton's condition factor was calculated for June 12, 2008 (= Condition Factor 1), August 28, 2008 (= Condition Factor 2), and November 14, 2008 (= Condition Factor 3), with the formula, K = [100 × BW × FL^-3^], where BW was body weight (g) and FL was fork length (cm). Body weight measurements obtained on August 28, 2008, and November 14, 2008 were designated as body weight 2 and body weight 3, respectively, in addition to the body weight 1 measurement made on June 12, 2008 (as mentioned above).

### Genetic marker analysis and map construction

Families 11, 18, and 28 exhibited high rates of early maturation (46-57%) and thus were not considered for genome scans. Variation coefficients for blood plasma osmolality (C_v _= 0.056-0.064) and Na^+^/K^+^-ATPase activity (C_v _= 0.33-0.40) were similar among the remaining three families (i.e., 9, 10, 12). Genetic maps were created for families 10 (n = 116) and 12 (n = 118), given that they exhibited lower early maturity than family 9, and thus provided a larger number of progeny for QTL analyses. Genetic markers were selected based on pre-existing Arctic charr linkage group assignments [28,31]. Where possible, markers were chosen at 20 cM intervals to ensure adequate QTL detection power [51] and comprehensive genome coverage. A standard phenol chloroform protocol was used for genomic DNA [52]. Forward or reverse marker primers were 5'-flourescently end-labeled with tetrachloro-6-carboxy-flourescent (TET) or 6-carboxy-floursecein (FAM). Polymerase chain reaction (PCR) mixtures were made in 7 μL volumes (2.6 ng genomic DNA·μL^-1^, 1× PCR buffer, 0.125 mM dNTP, 1.5-2.0 mM MgCl_2_, 0.1 mg·ml^-1 ^BSA, 0.3-0.6 μM of each primer, 0.021 U μL^-1 ^*Taq *DNA polymerase). PCR conditions began with initial denaturation (95°C for 10 min), followed by 35 cycles of denaturation (95°C for 1 min), annealing (50-58°C for 30 s), and extension (72°C for 30 s), and concluded with final extension (72°C for 5 min). Amplified PCR products were detected using polyacrylamide gel electrophoresis (6% polyacrylamide gel, 19:1 ratio of acrylamide to bisacrylamide, 8 M urea, 0.5× TBE buffer). Prior to loading, PCR products were mixed with 10 μl loading dye (95% formamide, 10 mM NaOH, 0.25% bromophenol blue) and denatured at 95°C for 10 min. Electrophoresis occurred under denaturing conditions in 1× TBE running buffer for 1.5-2.5 h at 1600 V. Gels were scanned using an FMBIO III Fluorescence scanner (MiraiBio Inc., Alameda, CA, USA).

Independent linkage maps were created for males and females due to large differences in recombination rates between the sexes [28,31,53]. Linkage of genetic markers and their relative order within linkage groups was established using several modules within the LINKMFEX software package (v2.3; LINKFMEX, LINKGRP, MAPORD, MAPDIS) [54]. Linkage was assigned based on a minimum logarithm of odds (LOD) score of 3.0.

### QTL analysis

Prior to QTL analysis all salinity tolerance traits were tested for deviation from normality (Kolmogorov-Smirnov and Lilliefors tests). Residual trait values (ANOVA) were used in QTL analyses as tank effects were detected independently in both families for all traits measured (i.e., blood plasma osmolality, Na^+^/K^+^-ATPase activity, specific growth rate 1, specific growth rate 2, body weight 1, body weight 2, body weight 3, condition factor 1, condition factor 2, and condition factor 3). Subsequent to the removal of tank effects a secondary effect of body weight 1 on Na^+^/K^+^-ATPase activity was detected in each family, therefore the residuals from a linear regression of Na^+^/K^+^-ATPase activity with body weight 1 were used in the QTL analysis for Na^+^/K^+^-ATPase activity. Statistics were performed with SYSTAT 12 for Windows (SYSTAT Software, Inc., 2007).

QTL analyses were based on 68 individuals per family for physiological QTL (i.e., Na^+^/K^+^-ATPase activity and blood plasma osmolality), and 112 individuals per family for body size and growth QTL. Linear regression-based interval analyses were conducted for each trait and parent independently using MultiQTL software (v2.5) [55]. Single-marker analysis was performed across all parents and families combined. Since interval distances among parental maps were often quite variable (i.e., large sex-specific differences in salmonid recombination rates), with random marker positions missing within the different parents used due to chance homozygous genotypes present in these outbred parents, it was not possible to perform the combined family analysis using interval analysis. For all QTL analyses, chromosome-wide LOD thresholds were determined empirically with 1000 permutations of the trait data against the genotypes [56]. Chromosome-wide significant QTL were assigned at a threshold of *P *≤ 0.05 (though QTL slightly above this threshold were also acknowledged), and then further tested for genome-wide significance using a B-H False Discovery Rate (FDR) test (α = 0.05).

## Authors' contributions

This study was conceptualized by MMF and RGD. The salinity tolerance trials and genome-scans were conducted by JDN, while JDN and RGD wrote the manuscript and performed the bioinformatics analyses. BG oversaw the rearing and maintenance of fish. All authors read and commented on the manuscript.

## Supplementary Material

Additional file 1 **Genetic linkage map for family 10 female**. Click here for file

Additional file 2 **Genetic linkage map for family 10 male**. Click here for file

Additional file 3 **Genetic linkage map for family 12 female**. Click here for file

Additional file 4 **Genetic linkage map for family 12 male**. Click here for file

Additional file 5 **QTL for Na^+^/K^+^-ATPase activity and blood plasma osmolality in two Arctic charr (*Salvelinus alpinus*) full-sib families**. Click here for file

Additional file 6 **QTL for growth in sea water in two Arctic charr (*Salvelinus alpinus*) full-sib families**. Click here for file

Additional file 7 **QTL for salinity tolerance traits based on a combined analysis of two Arctic charr (*Salvelinus alpinus*) full-sib families**. Click here for file

Additional file 8 **QTL homeologies for seawater and body size traits in Arctic charr (*Salvelinus alpinus*)**. Click here for file

Additional file 9 **QTL for body weight and Fulton's condition factor in two Arctic charr (*Salvelinus alpinus*) full-sib families**. Click here for file

Additional file 10 **QTL for body weight and condition factor based on a combined analysis of two Arctic charr (*Salvelinus alpinus*) full-sib families**. Click here for file

## References

[B1] Marshall W Grosell M Evans D, Claiborne J Ion transport, osmoregulation, and acid-base balance The physiology of fishes 2006 3 Boca Raton, FL: CRC Press 177 230

[B2] Silva P Solomon R Spokes K Epstein F Ouabain inhibition of gill Na-K-ATPase: relationship to active chloride transport J Exp Zool 1977 199 419 426 10.1002/jez.1401990316 139454

[B3] Furuse M Furuse K Sasaki H Tsukita S Conversion of Zonulae occludentes from tight to leaky strand type by introducing claudin-2 into Madin-Darby canine kidney I cells J Cell Biol 2001 153 2 263 272 10.1083/jcb.153.2.263 11309408 PMC2169456

[B4] Tipsmark CK Kiilerich P Nilsen TO Ebbesson LOE Stefansson SO Madsen SS Branchial expression patterns of claudin isoforms in Atlantic salmon during seawater acclimation and smoltification Am J Physiol-Reg I 2008 294 5 R1563 R1574 10.1152/ajpregu.00915.2007 18321951

[B5] Van Itallie CM Rogan S Yu A Vidal LS Holmes J Anderson JM Two splice variants of claudin-10 in the kidney create paracellular pores with different ion selectivities Am J Physiol-Renal 2006 291 6 F1288 F1299 10.1152/ajprenal.00138.2006 16804102

[B6] Richards JG Semple JW Bystriansky JS Schulte PM Na^+^/K^+^-ATPase (alpha-isoform switching in gills of rainbow trout (Oncorhynchus mykiss) during salinity transfer J Exp Biol 2003 206 24 4475 4486 10.1242/jeb.00701 14610032

[B7] Bystriansky JS Richards JG Schulte PM Ballantyne JS Reciprocal expression of gill Na^+^/K^+^-ATPase alpha-subunit isoforms alpha 1a and alpha 1b during seawater acclimation of three salmonid fishes that vary in their salinity tolerance J Exp Biol 2006 209 10 1848 1858 10.1242/jeb.02188 16651551

[B8] Mackie P Wright PA Glebe BD Ballantyne JS Osmoregulation and gene expression of Na^+^/K^+ ^ATPase in families of Atlantic salmon (*Salmo salar*) smolts Can J Fish Aqua Sci 2005 62 11 2661 2672 10.1139/f05-168

[B9] McCormick SD Regish AM Christensen AK Distinct freshwater and seawater isoforms of Na(+)/K(+)-ATPase in gill chloride cells of Atlantic salmon J Exp Biol 2009 212 24 3994 4001 10.1242/jeb.037275 19946077

[B10] Larsen PF Nielsen EE Koed A Thomsen DS Olsvik PA Loeschcke V Interpopulation differences in expression of candidate genes for salinity tolerance in winter migrating anadromous brown trout (*Salmo trutta *L.) BMC Genet 2008 9 12 18230136 10.1186/1471-2156-9-12 PMC2254441

[B11] Schmitz M Seasonal-changes in hypoosmoregulatory ability in landlocked and anadromous populations of Arctic charr, *Salvelinus alpinus*, and Atlantic salmon, *Salmo salar* Environ Biol Fish 1995 42 4 401 412 10.1007/BF00001471

[B12] Singer TD Clements KM Semple JW Schulte PM Bystriansky JS Finstad B Fleming IA McKinley RS Seawater tolerance and gene expression in two strains of Atlantic salmon smolts Can J Fish Aquat Sci 2002 59 1 125 135 10.1139/f01-205

[B13] Shrimpton JM Patterson DA Richards JG Cooke SJ Schulte PM Hinch SG Farrell AP Ionoregulatory changes in different populations of maturing sockeye salmon *Oncorhynchus nerka *during ocean and river migration J Exp Biol 2005 208 21 4069 4078 10.1242/jeb.01871 16244166

[B14] Nilsen TO Ebbesson LOE Madsen SS McCormick SD Andersson E Bjornsson BT Prunet P Stefansson SO Differential expression of gill Na+,K+-ATPase alpha- and beta-subunits, Na+,K+,2Cl(-) cotransporter and CFTR anion channel in juvenile anadromous and landlocked Atlantic salmon *Salmo salar* J Exp Biol 2007 210 16 2885 2896 10.1242/jeb.002873 17690237

[B15] Hoar W Hoar W, Randall D The physiology of smolting salmonids Fish Physiology 1988 XIV New York: Academic Press 275 343

[B16] Hiroi J McCormick SD Variation in salinity tolerance, gill Na^+^/K^+^-ATPase, Na^+^/K^+^/2Cl(-) cotransporter and mitochondria-rich cell distribution in three salmonids *Salvelinus namaycush, Salvelinus fontinalis *and *Salmo salar* J Exp Biol 2007 210 6 1015 1024 10.1242/jeb.002030 17337714

[B17] Delabbio JL Glebe BD Sreedharan A Variation in growth and survival between 2 anadromous strains of Canadian Arctic charr (*Salvelinus alpinus*) during long-term saltwater rearing Aquaculture 1990 85 1-4 259 270 10.1016/0044-8486(90)90025-I

[B18] Nielsen C Holdensaard G Petersen HC Bjornsson BT Madsen SS Genetic differences in physiology, growth hormone levels and migratory behaviour of Atlantic salmon smolts J Fish Biol 2001 59 1 28 44 10.1111/j.1095-8649.2001.tb02336.x

[B19] Boula D Castric V Bernatchez L Audet C Physiological, endocrine, and genetic bases of anadromy in the brook charr, *Salvelinus fontinalis*, of the Laval River (Quebec, Canada) Environ Biol Fish 2002 64 1-3 229 242

[B20] Handeland SO Bjornsson BT Arnesen AM Stefansson SO Seawater adaptation and growth of post-smolt Atlantic salmon (*Salmo salar*) of wild and farmed strains Aquaculture 2003 220 1-4 367 384 10.1016/S0044-8486(02)00508-2

[B21] Le Bras Y Dechamp N Krieg F Filangi O Guyomard R Boussaha M Bovenhuis H Pottinger TG Prunet P Le Roy P Detection of QTL with effects on osmoregulation capacities in the rainbow trout (*Oncorhynchus mykiss*) BMC Genet 2011 12 46 21569550 10.1186/1471-2156-12-46 PMC3120726

[B22] Spring J Vertebrate evolution by interspecific hybridisation - Are we polyploid? Febs Lett 1997 400 1 2 8 10.1016/S0014-5793(96)01351-8 9000502

[B23] Jaillon O Aury JM Brunet F Petit JL Stange-Thomann N Mauceli E Bouneau L Fischer C Ozouf-Costaz C Bernot A Genome duplication in the teleost fish *Tetraodon nigroviridis *reveals the early vertebrate proto-karyotype Nature 2004 431 7011 946 957 10.1038/nature03025 15496914

[B24] Allendorf F Thorgaard G BJ T Tetraploidy and the evolution of salmonid fishes Evolutionary genetics of fishes 1984 New York: Plenum Press 1 46

[B25] Allendorf FW Danzmann RG Secondary tetrasomic segregation of MDH-B and preferential pairing of homeologues in rainbow trout Genetics 1997 145 4 1083 1092 9093860 10.1093/genetics/145.4.1083 PMC1207878

[B26] Gharbi K Ferguson MM Danzmann RG Characterization of Na, K-ATPase genes in Atlantic salmon (*Salmo salar*) and comparative genomic organization with rainbow trout (*Oncorhynchus mykiss*) Mol Genet Genomics 2005 273 6 474 483 10.1007/s00438-005-1135-8 15883826

[B27] Gharbi K Semple JW Ferguson MM Schulte PM Danzmann RG Linkage arrangement of Na,K-ATPase genes in the tetraploid-derived genome of the rainbow trout (*Oncorhynchus mykiss*) Anim Genet 2004 35 4 321 325 10.1111/j.1365-2052.2004.01152.x 15265073

[B28] Danzmann RG Cairney M Davidson WS Ferguson MM Gharbi K Guyomard R Holm LE Leder E Okamoto N Ozaki A A comparative analysis of the rainbow trout genome with 2 other species of fish (Arctic charr and Atlantic salmon) within the tetraploid derivative Salmonidae family (subfamily: Salmoninae) Genome 2005 48 6 1037 1051 10.1139/g05-067 16391673

[B29] Timusk E Ferguson M Moghadam H Norman J Wilson C Danzmann R Genome evolution in the fish family Salmonidae: generation of a brook charr genetic map and comparisons among charrs (Arctic charr and brook charr) with rainbow trout BMC Genet 2011 12 68 21798024 10.1186/1471-2156-12-68 PMC3162921

[B30] Nichols KM Edo AF Wheeler PA Thorgaard GH The genetic basis of smoltification-related traits in *Oncorhynchus mykiss* Genetics 2008 179 3 1559 1575 10.1534/genetics.107.084251 18562654 PMC2475755

[B31] Woram RA McGowan C Stout JA Gharbi K Ferguson MM Hoyheim B Davidson EA Davidson WS Rexroad C Danzmann RG A genetic linkage map for Arctic char (*Salvelinus alpinus*): evidence for higher recombination rates and segregation distortion in hybrid versus pure strain mapping parents Genome 2004 47 2 304 315 10.1139/g03-127 15060583

[B32] Danzmann RG Davidson EA Ferguson MM Gharbi K Koop BF Hoyheim B Lien S Lubieniecki KP Moghadam HK Park J Distribution of ancestral proto-Actinopterygian chromosome arms within the genomes of 4R-derivative salmonid fishes (Rainbow trout and Atlantic salmon) BMC Genomics 2008 9 16 10.1186/1471-2164-9-16 19032764 PMC2632648

[B33] Cutler CP Cramb G Two isoforms of the Na+/K+/2CI(-) cotransporter are expressed in the European eel (*Anguilla anguilla*) BBA-Biomembranes 2002 1566 1-2 92 103 10.1016/S0005-2736(02)00596-5 12421541

[B34] Chen JM Cutler C Jacques C Boeuf G Denamur E Lecointre G Mercier B Cramb G Ferec C A combined analysis of the cystic fibrosis transmembrane conductance regulator: Implications for structure and disease models Mol Biol Evol 2001 18 9 1771 1788 11504857 10.1093/oxfordjournals.molbev.a003965

[B35] Moghadam HK Ferguson MM Rexroad CE Coulibaly I Danzmann RG Genomic organization of the IGF1, IGF2, MYF5, MYF6 and GRF/PACAP genes across Salmoninae genera Anim Genet 2007 2007 38 5 527 532 10.1111/j.1365-2052.2007.01645.x 17894566

[B36] Shamblott MJ Cheng CM Bolt D Chen TT Appearance of inslulin-like growth factor messanger RNA in the liver and pyloric ceca of a teleost in response to exogenous growth hormone P Natl A Sci USA 1995 92 15 6943 6946 10.1073/pnas.92.15.6943 PMC41447 7624349

[B37] Bolton JP Collie NL Kawauchi H Hirano T Osmoregulatory actions of growht hormone in rainbow trout (*Salmo gairdneri*) J Endocrinol 1987 112 1 63 68 10.1677/joe.0.1120063 3819633

[B38] McCormick SD Endocrine control of osmoregulation in teleost fish Am Zool 2001 41 4 781 794 10.1668/0003-1569(2001)041[0781:ECOOIT]2.0.CO;2

[B39] Pelis RM McCormick SD Effects of growth hormone and cortisol on Na+-K+-2Cl(-) cotransporter localization and abundance in the gills of Atlantic salmon Gen Comp Endocr 2001 124 2 134 143 10.1006/gcen.2001.7703 11703079

[B40] Boutet I Ky CLL Bonhomme F A transcriptomic approach of salinity response in the euryhaline teleost, *Dicentrarchus labrax* Gene 2006 379 40 50 16737785 10.1016/j.gene.2006.04.011

[B41] Kalujnaia S McWilliam IS Zaguinaiko VA Feilen AL Nicholson J Hazon N Cutler CP Cramb G Transcriptomic approach to the study of osmoregulation in the European eel *Anguilla anguilla* Physiol Genomics 2007 31 3 385 401 10.1152/physiolgenomics.00059.2007 17666525

[B42] Evans TG Somero GN A microarray-based transcriptomic time-course of hyper- and hypo-osmotic stress signaling events in the euryhaline fish *Gillichthys mirabilis*: osmosensors to effectors J Exp Biol 2008 211 22 3636 3649 10.1242/jeb.022160 18978229

[B43] Tine M de Lorgeril J D'Cotta H Pepey E Bonhomme F Baroiller JF Durand J-D Transcriptional responses of the black-chinned tilapia *Sarotherodon melanotheron *to salinity extremes Mar Genomics 2008 1 2 37 46 10.1016/j.margen.2008.06.001 21798152

[B44] Link K Berishvili G Shved N D'Cotta H Baroiller J-F Reinecke M Eppler E Seawater and freshwater challenges affect the insulin-like growth factors IGF-I and IGF-II in liver and osmoregulatory organs of the tilapia Mol Cell Endocrinol 2010 327 1-2 40 46 10.1016/j.mce.2010.05.011 20621706

[B45] Moghadam HK Poissant J Fotherby H Haidle L Ferguson MM Danzmann RG Quantitative trait loci for body weight, condition factor and age at sexual maturation in Arctic charr (*Salvelinus alpinus*): comparative analysis with rainbow trout (*Oncorhynchus mykiss*) and Atlantic salmon (*Salmo salar*) Mol Genet Genomics 2007 277 6 647 661 10.1007/s00438-007-0215-3 17308931

[B46] Mackay TFC The genetic architecture of quantitative traits Annu Rev Genet 2001 35 303 339 10.1146/annurev.genet.35.102401.090633 11700286

[B47] Cote G Perry G Blier P Bernatchez L The influence of gene-environment interactions on GHR and IGF-I expression and their association with growth in brook charr, *Salvelinus fontinalis *(Mitchill) BMC Genet 2007 8 87 18154679 10.1186/1471-2156-8-87 PMC2257973

[B48] Martyniuk CJ Perry GML Mogahadam HK Ferguson MM Danzmann RG The genetic architecture of correlations among growth-related traits and male age at maturation in rainbow trout J Fish Biol 2003 63 3 746 764 10.1046/j.1095-8649.2003.00188.x

[B49] McCormick SD Methods for non-lethal gill biopsy and measurement of Na^+^,K^+^-ATPase activity Can J Fish Aquat Sci 1993 50 3 656 658 10.1139/f93-075

[B50] Ricker W Hoar W, Randall D, Brett J Growth rates and models Fish Physiology 1979 VIII New York: Academic Press

[B51] Darvasi A Weinreb A Minke V Weller JI Soller M Detecting marker-QTL linkage and estimating gene effect and map location using a saturated genetic map Genetics 1993 134 3 943 951 8349116 10.1093/genetics/134.3.943 PMC1205528

[B52] Taggart JB Hynes RA Prodohl PA Ferguson A A simplified protocol for routine total DNA isolation from salmonid fishes J Fish Biol 1992 40 6 963 965 10.1111/j.1095-8649.1992.tb02641.x

[B53] Sakamoto T Danzmann RG Gharbi K Howard P Ozaki A Khoo SK Woram RA Okamoto N Ferguson MM Holm LE A microsatellite linkage map of rainbow trout (*Oncorhynchus mykiss*) characterized by large sex-specific differences in recombination rates Genetics 2000 155 3 1331 1345 10880492 10.1093/genetics/155.3.1331 PMC1461176

[B54] Faculty webpage at the University of Guelph http://www.uoguelph.ca/~rdanzman/software.htm

[B55] MultiQTL Home Page http://www.multiqtl.com

[B56] Churchill GA Doerge RW Empirical threshold values for quantitative trait mapping Genetics 1994 138 3 963 971 7851788 10.1093/genetics/138.3.963 PMC1206241

