# Supplementary material for: The genetic basis of salinity tolerance traits in Arctic charr (Salvelinus alpinus)
Source: BMC Genet. 2011 Sep 21;12:81. doi: 10.1186/1471-2156-12-81 (PMC3190344; doi:10.1186/1471-2156-12-81)
Supplement: Additional file 1 — Genetic linkage map for family 10 female. [file 1471-2156-12-81-S1.PDF]

Additional File 1 - Linkage Map for Family 10 Female

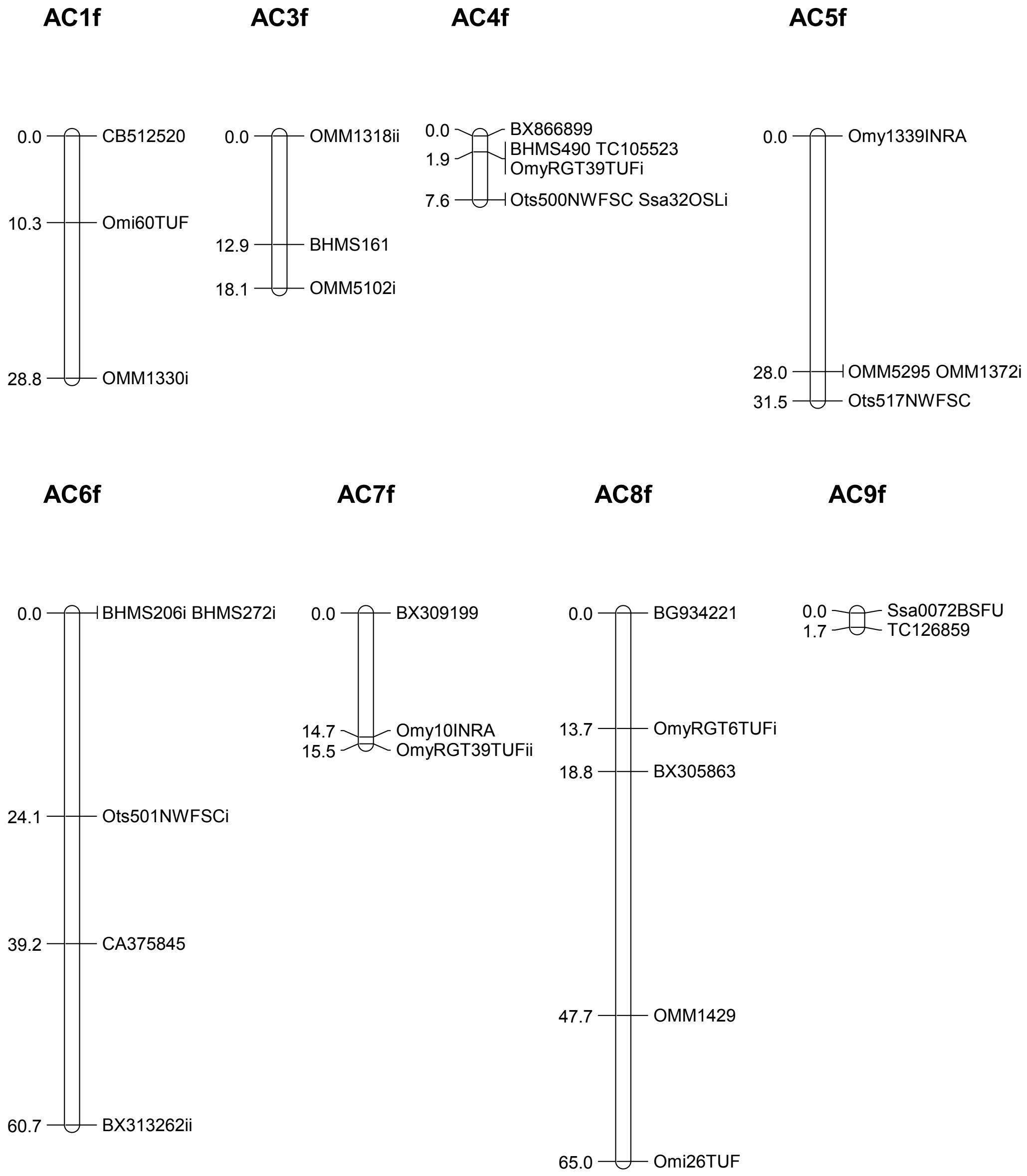

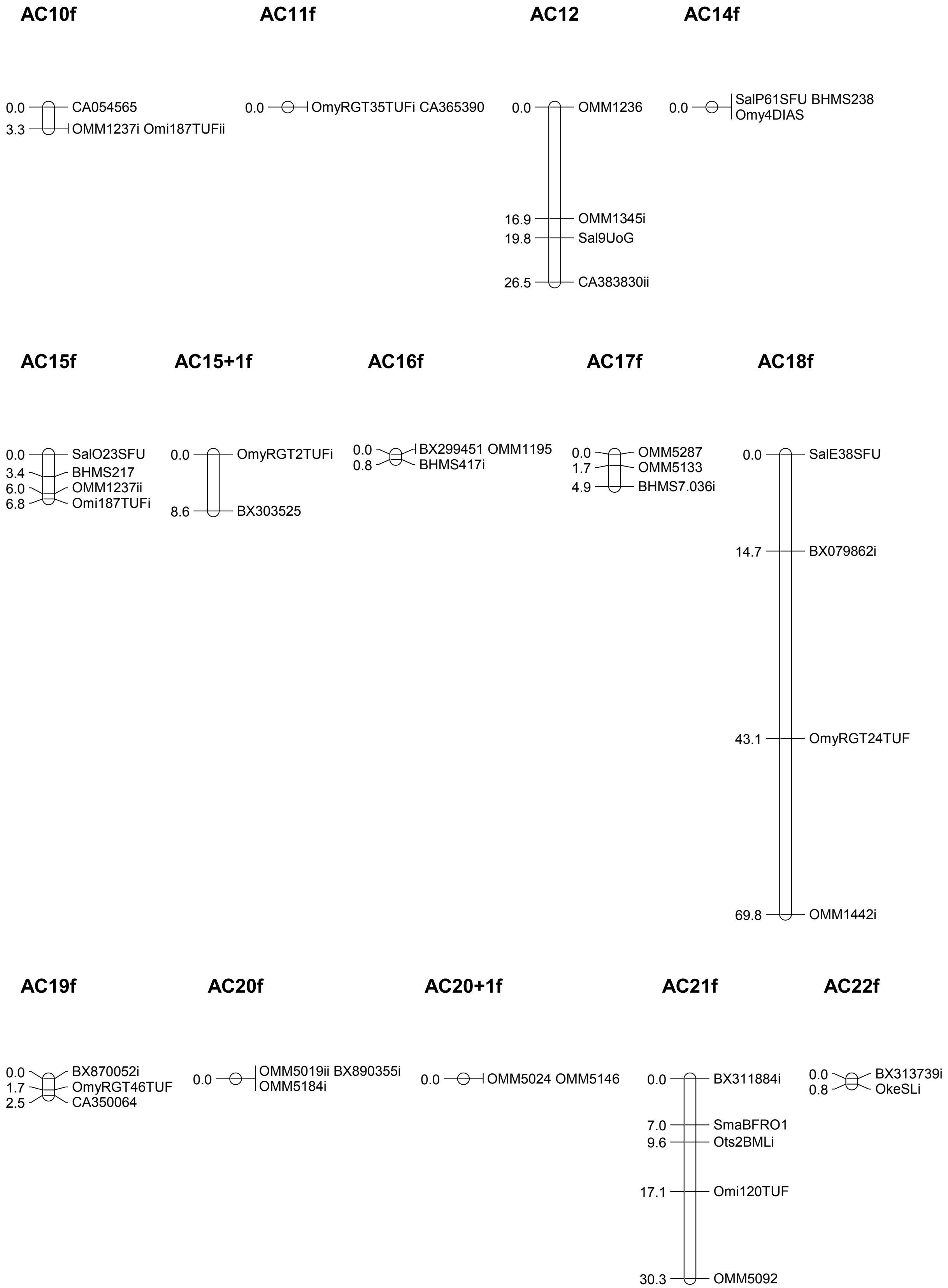
AC10f
0.0 CA054565
3.3 OMM1237i Omi187TUFii
AC11f
0.0 OmyRGT35TUFi CA365390
AC12
0.0 OMM1236
16.9 OMM1345i
19.8 Sal9UoG
26.5 CA383830ii
AC14f
0.0 SalP61SFU BHMS238 Omy4DIAS
AC15f
0.0 SalO23SFU
3.4 BHMS217
6.0 OMM1237ii
6.8 Omi187TUFi
AC15+1f
0.0 OmyRGT2TUFi
8.6 BX303525
AC16f
0.0 BX299451 OMM1195
0.8 BHMS417i
AC17f
0.0 OMM5287
1.7 OMM5133
4.9 BHMS7.036i
AC18f
0.0 SalE38SFU
14.7 BX079862i
43.1 OmyRGT24TUF
69.8 OMM1442i
AC19f
0.0 BX870052i
1.7 OmyRGT46TUF
2.5 CA350064
AC20f
0.0 OMM5019ii BX890355i OMM5184i
AC20+1f
0.0 OMM5024 OMM5146
AC21f
0.0 BX311884i
7.0 SmaBFRO1
9.6 Ots2BMLi
17.1 Omi120TUF
30.3 OMM5092
AC22f
0.0 BX313739i
0.8 OkeSLi

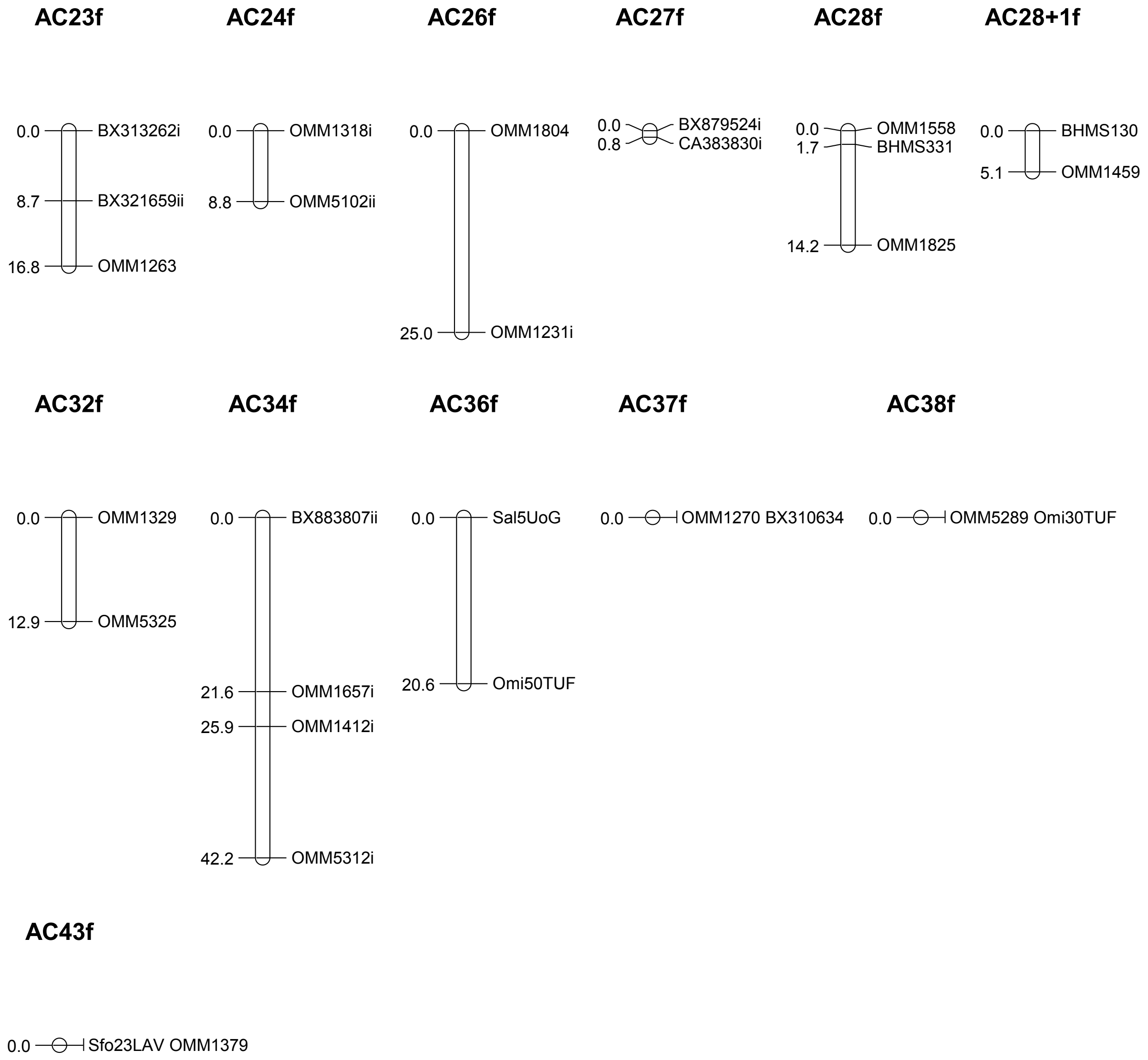
AC23f
0.0 BX313262i
8.7 BX321659ii
16.8 OMM1263
AC24f
0.0 OMM1318i
8.8 OMM5102ii
AC26f
0.0 OMM1804
25.0 OMM1231i
AC27f
0.0 BX879524i
0.8 CA383830i
AC28f
0.0 OMM1558
1.7 BHMS331
14.2 OMM1825
AC28+1f
0.0 BHMS130
5.1 OMM1459
AC32f
0.0 OMM1329
12.9 OMM5325
AC34f
0.0 BX883807ii
21.6 OMM1657i
25.9 OMM1412i
42.2 OMM5312i
AC36f
0.0 Sal5UoG
20.6 Omi50TUF
AC37f
0.0 OMM1270 BX310634
AC38f
0.0 OMM5289 Omi30TUF
AC43f
0.0 Sfo23LAV OMM1379
