# Supplementary material for: The genetic basis of salinity tolerance traits in Arctic charr (Salvelinus alpinus)
Source: BMC Genet. 2011 Sep 21;12:81. doi: 10.1186/1471-2156-12-81 (PMC3190344; doi:10.1186/1471-2156-12-81)
Supplement: Additional file 2 — Genetic linkage map for family 10 male. [file 1471-2156-12-81-S2.PDF]

# Additional File 2 - Linkage Map for Family 10 Male

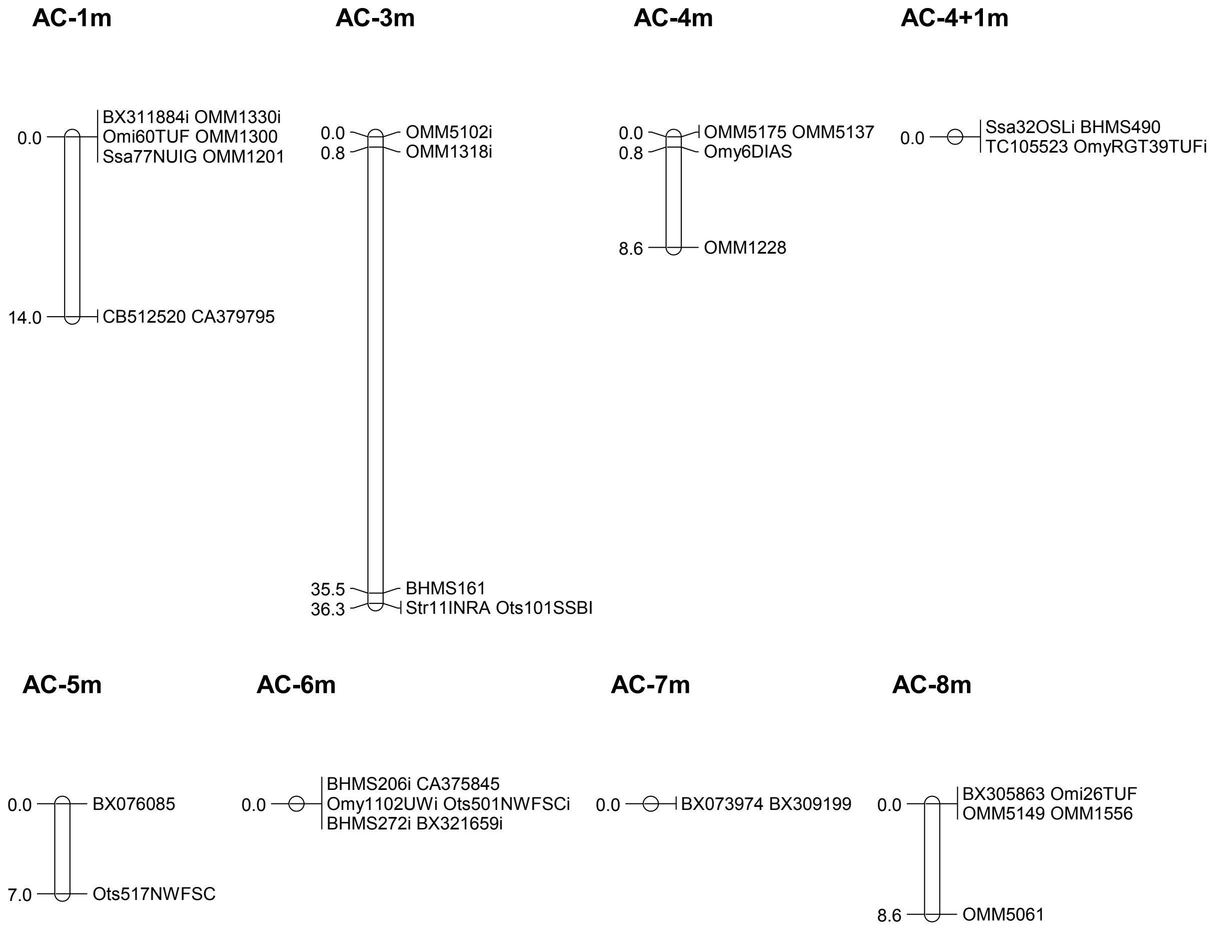

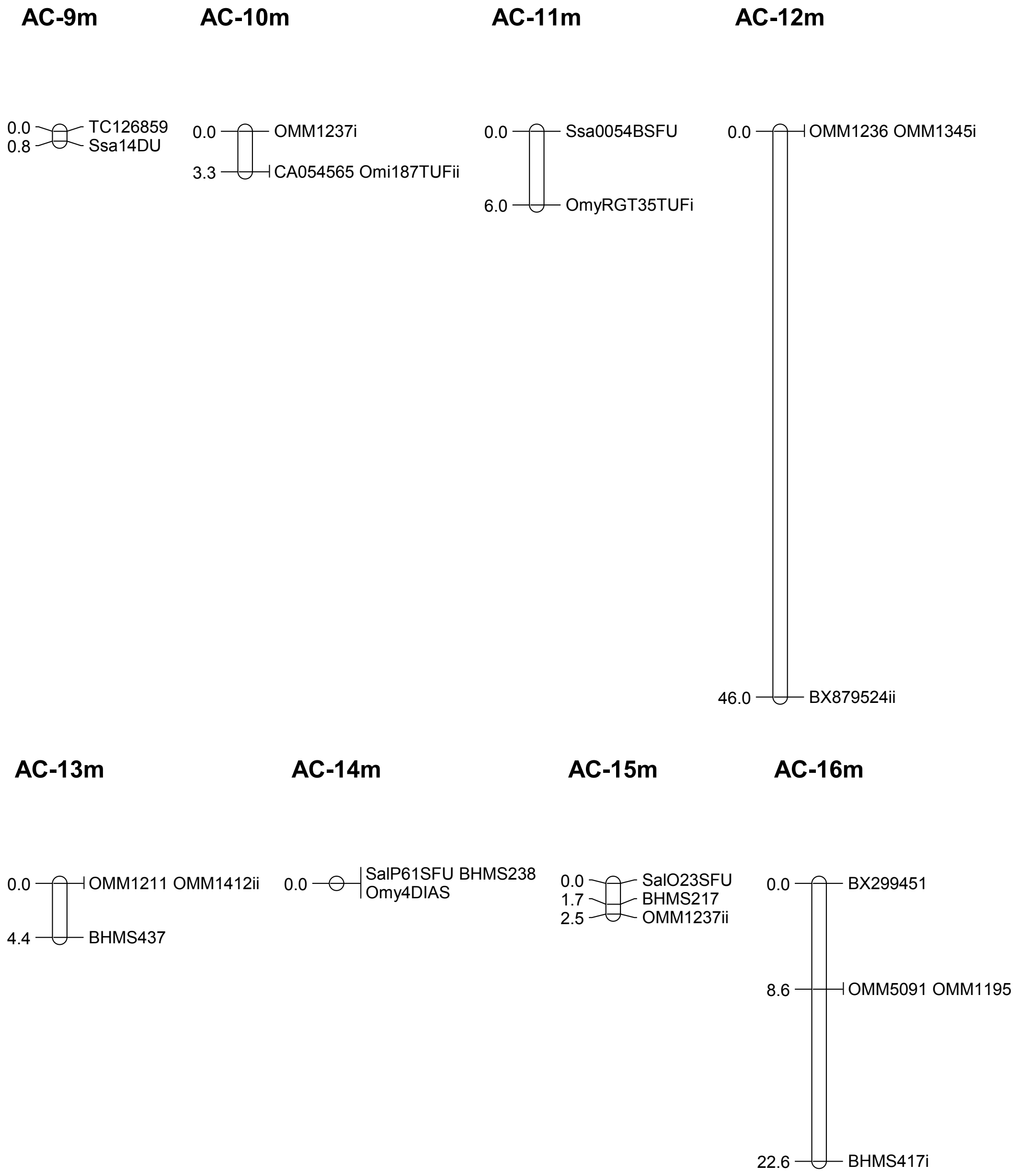
AC-9m
0.0 TC126859
0.8 Ssa14DU
AC-10m
0.0 OMM1237i
3.3 CA054565 Omi187TUFii
AC-11m
0.0 Ssa0054BSFU
6.0 OmyRGT35TUFi
AC-12m
0.0 OMM1236 OMM1345i
46.0 BX879524ii
AC-13m
0.0 OMM1211 OMM1412ii
4.4 BHMS437
AC-14m
0.0 SalP61SFU BHMS238
Omy4DIAS
AC-15m
0.0 SalO23SFU
1.7 BHMS217
2.5 OMM1237ii
AC-16m
0.0 BX299451
8.6 OMM5091 OMM1195
22.6 BHMS417i

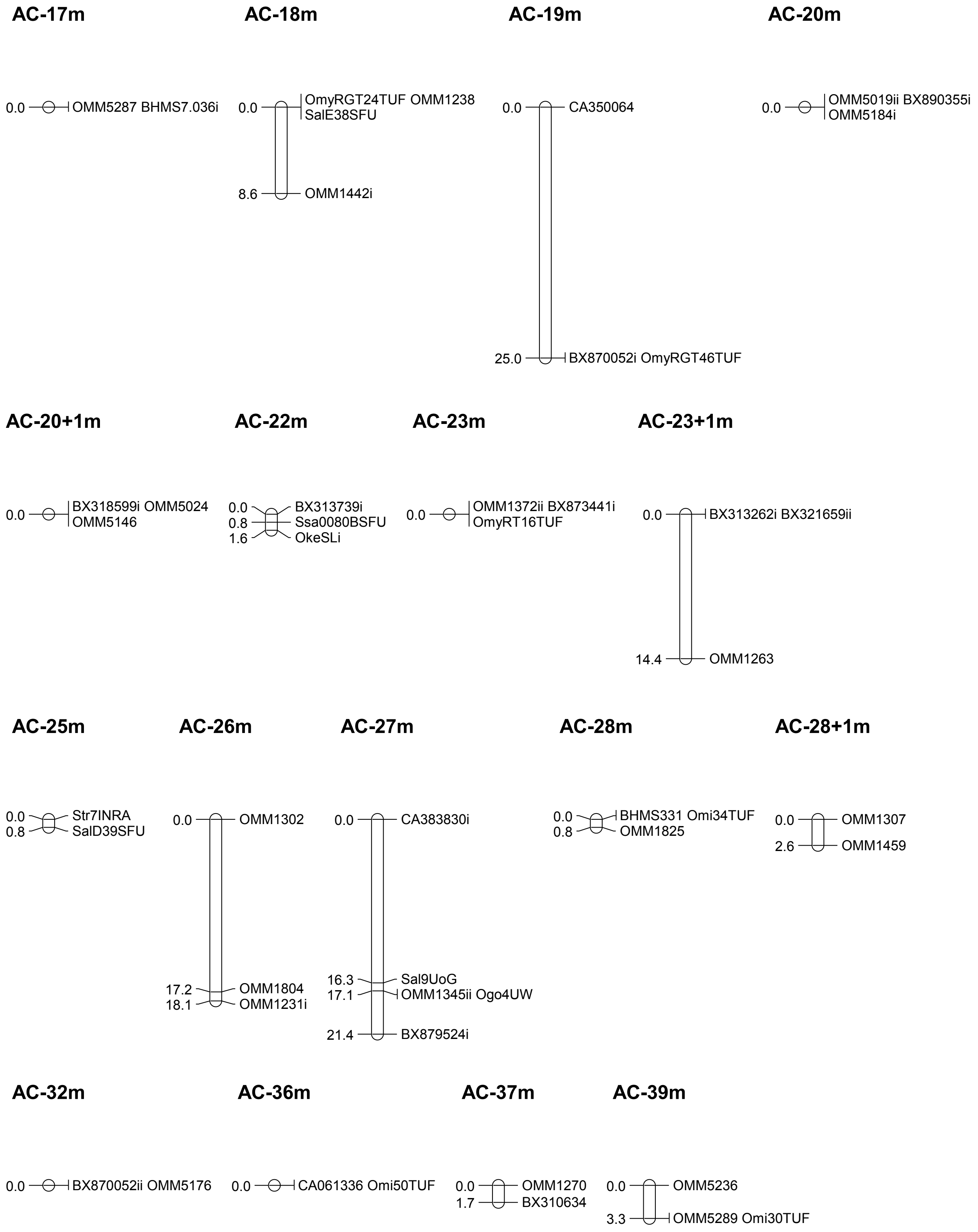
AC-17m
0.0 OMM5287 BHMS7.036i
AC-18m
0.0 OmyRGT24TUF OMM1238
SalE38SFU
8.6 OMM1442i
AC-19m
0.0 CA350064
25.0 BX870052i OmyRGT46TUF
AC-20m
0.0 OMM5019ii BX890355i
OMM5184i
AC-20+1m
0.0 BX318599i OMM5024
OMM5146
AC-22m
0.0 BX313739i
0.8 Ssa0080BSFU
1.6 OkeSLi
AC-23m
0.0 OMM1372ii BX873441i
OmyRT16TUF
AC-23+1m
0.0 BX313262i BX321659ii
14.4 OMM1263
AC-25m
0.0 Str7INRA
0.8 SalD39SFU
AC-26m
0.0 OMM1302
17.2 OMM1804
18.1 OMM1231i
AC-27m
0.0 CA383830i
16.3 Sal9UoG
17.1 OMM1345ii Ogo4UW
21.4 BX879524i
AC-28m
0.0 BHMS331 Omi34TUF
0.8 OMM1825
AC-28+1m
0.0 OMM1307
2.6 OMM1459
AC-32m
0.0 BX870052ii OMM5176
AC-36m
0.0 CA061336 Omi50TUF
AC-37m
0.0 OMM1270
1.7 BX310634
AC-39m
0.0 OMM5236
3.3 OMM5289 Omi30TUF

**AC-43m**

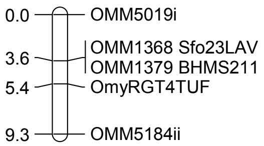
0.0
OMM5019i
3.6
OMM1368 Sfo23LAV
OMM1379 BHMS211
5.4
OmyRGT4TUF
9.3
OMM5184ii
