# Supplementary material for: The genetic basis of salinity tolerance traits in Arctic charr (Salvelinus alpinus)
Source: BMC Genet. 2011 Sep 21;12:81. doi: 10.1186/1471-2156-12-81 (PMC3190344; doi:10.1186/1471-2156-12-81)
Supplement: Additional file 3 — Genetic linkage map for family 12 female. [file 1471-2156-12-81-S3.PDF]

# Additional File 3 - Linkage Map for Family 12 Female

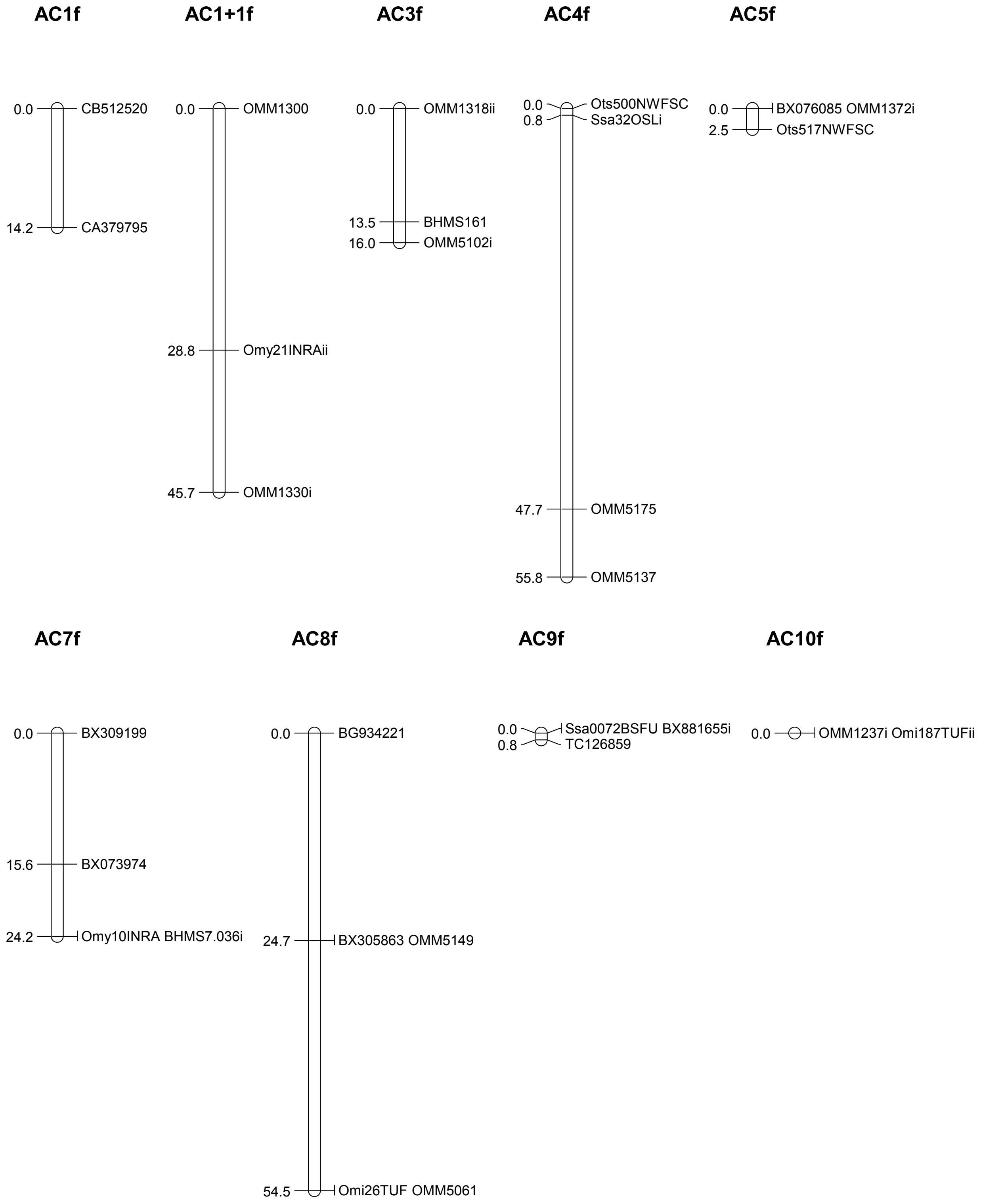

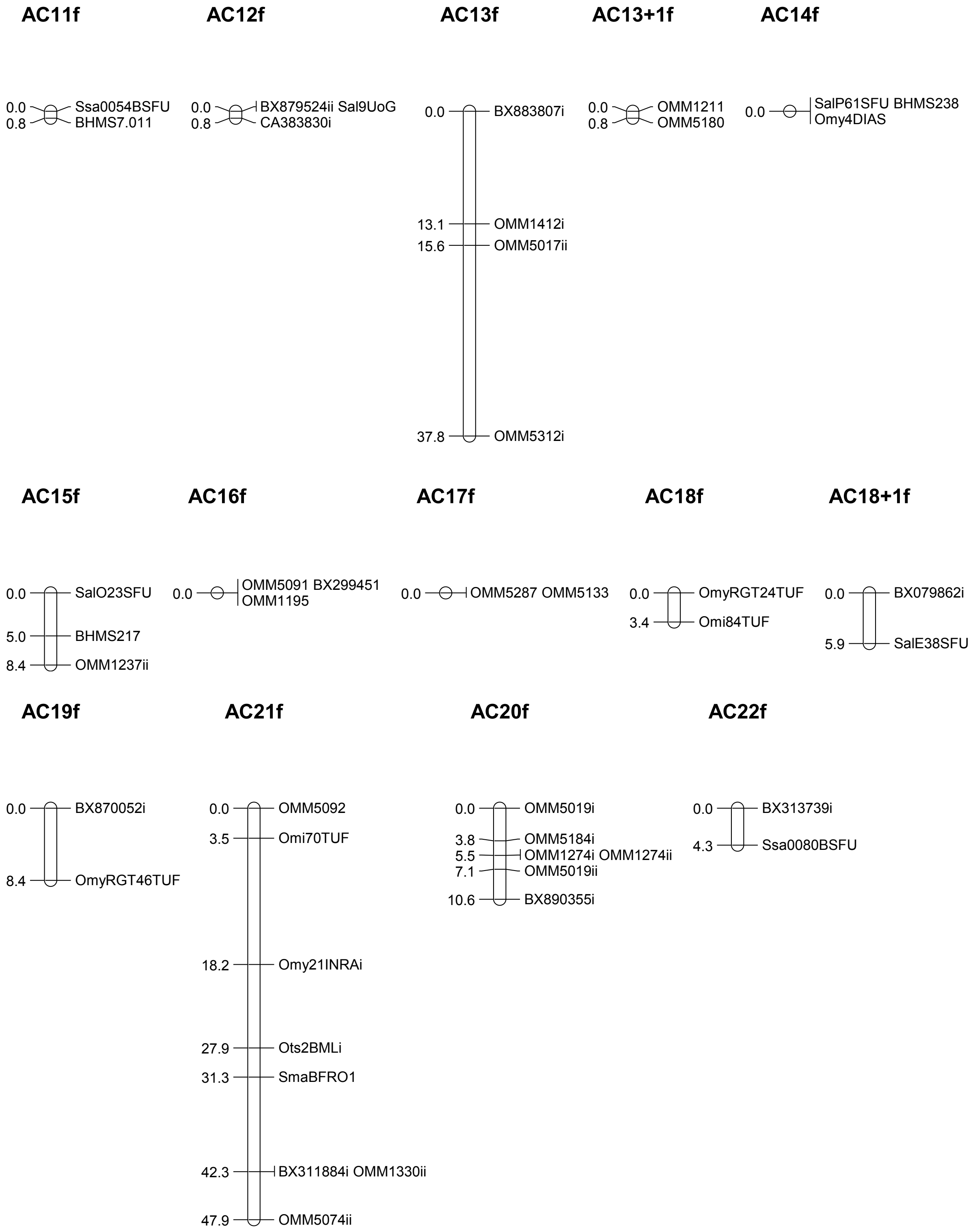

AC11f
0.0 Ssa0054BSFU
0.8 BHMS7.011
AC12f
0.0 BX879524ii Sal9UoG
0.8 CA383830i
AC13f
0.0 BX883807i
13.1 OMM1412i
15.6 OMM5017ii
37.8 OMM5312i
AC13+1f
0.0 OMM1211
0.8 OMM5180
AC14f
0.0 SalP61SFU BHMS238 Omy4DIAS
AC15f
0.0 SalO23SFU
5.0 BHMS217
8.4 OMM1237ii
AC16f
0.0 OMM5091 BX299451 OMM1195
AC17f
0.0 OMM5287 OMM5133
AC18f
0.0 OmyRGT24TUF
3.4 Omi84TUF
AC18+1f
0.0 BX079862i
5.9 SalE38SFU
AC19f
0.0 BX870052i
8.4 OmyRGT46TUF
AC21f
0.0 OMM5092
3.5 Omi70TUF
18.2 Omy21INRAi
27.9 Ots2BMLi
31.3 SmaBFRO1
42.3 BX311884i OMM1330ii
47.9 OMM5074ii
AC20f
0.0 OMM5019i
3.8 OMM5184i
5.5 OMM1274i OMM1274ii
7.1 OMM5019ii
10.6 BX890355i
AC22f
0.0 BX313739i
4.3 Ssa0080BSFU

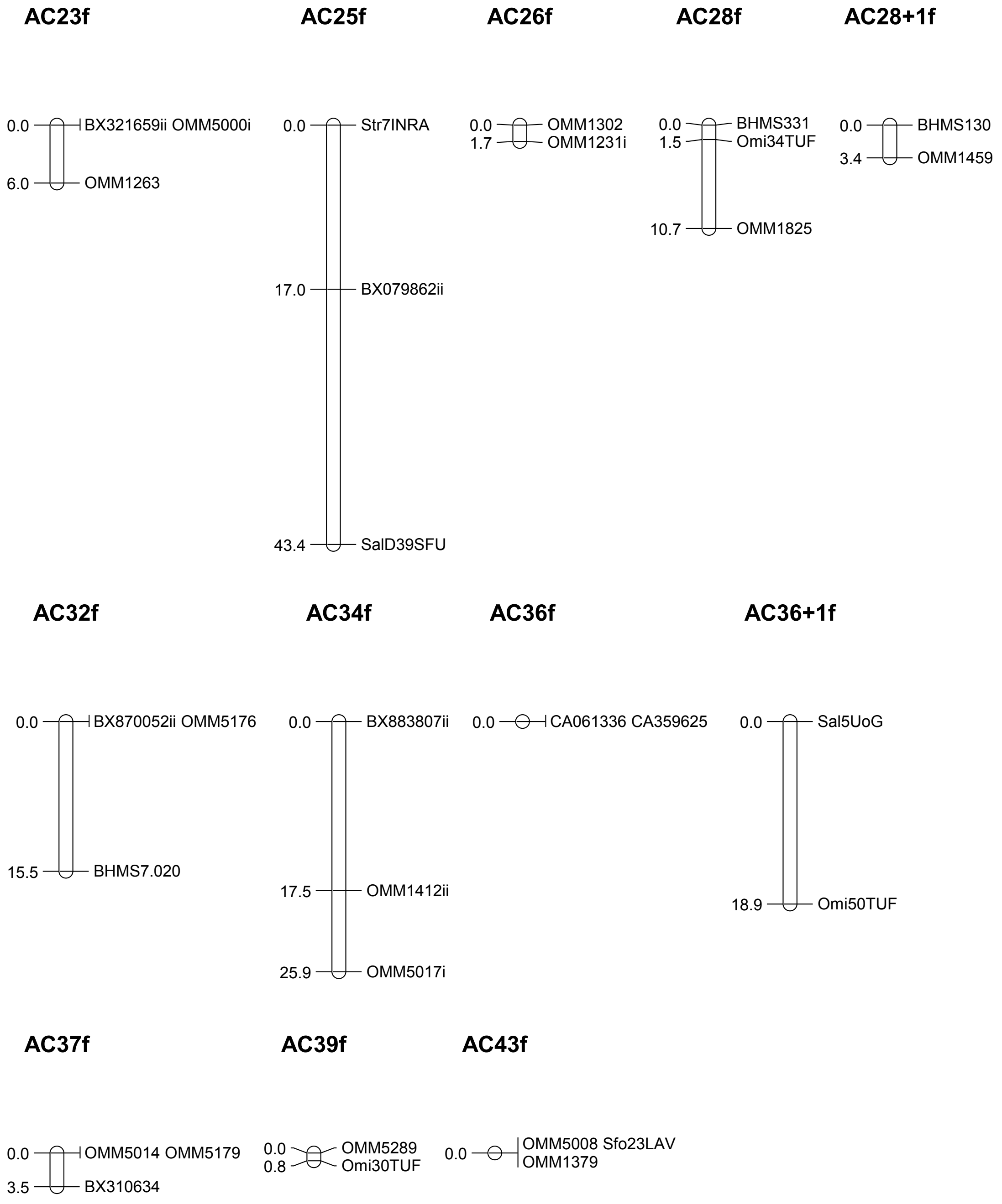
AC23f
0.0 BX321659ii OMM5000i
6.0 OMM1263
AC25f
0.0 Str7INRA
17.0 BX079862ii
43.4 SalD39SFU
AC26f
0.0 OMM1302
1.7 OMM1231i
AC28f
0.0 BHMS331
1.5 Omi34TUF
10.7 OMM1825
AC28+1f
0.0 BHMS130
3.4 OMM1459
AC32f
0.0 BX870052ii OMM5176
15.5 BHMS7.020
AC34f
0.0 BX883807ii
17.5 OMM1412ii
25.9 OMM5017i
AC36f
0.0 CA061336 CA359625
AC36+1f
0.0 Sal5UoG
18.9 Omi50TUF
AC37f
0.0 OMM5014 OMM5179
3.5 BX310634
AC39f
0.0 OMM5289
0.8 Omi30TUF
AC43f
0.0 OMM5008 Sfo23LAV
OMM1379
