# Supplementary material for: The genetic basis of salinity tolerance traits in Arctic charr (Salvelinus alpinus)
Source: BMC Genet. 2011 Sep 21;12:81. doi: 10.1186/1471-2156-12-81 (PMC3190344; doi:10.1186/1471-2156-12-81)
Supplement: Additional file 4 — Genetic linkage map for family 12 male. [file 1471-2156-12-81-S4.PDF]

# Additional File 4 - Linkage Map for Family 12 Male

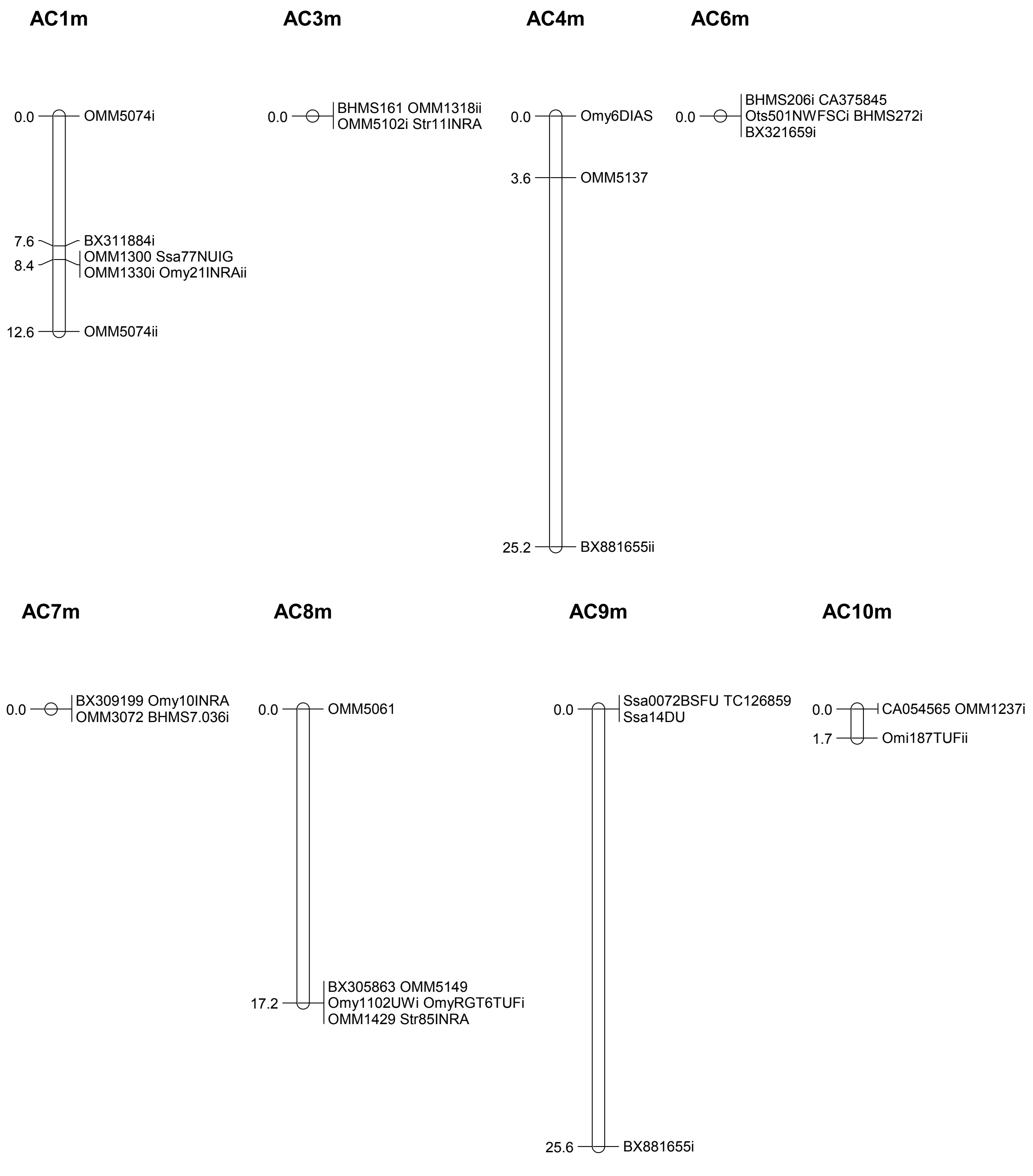

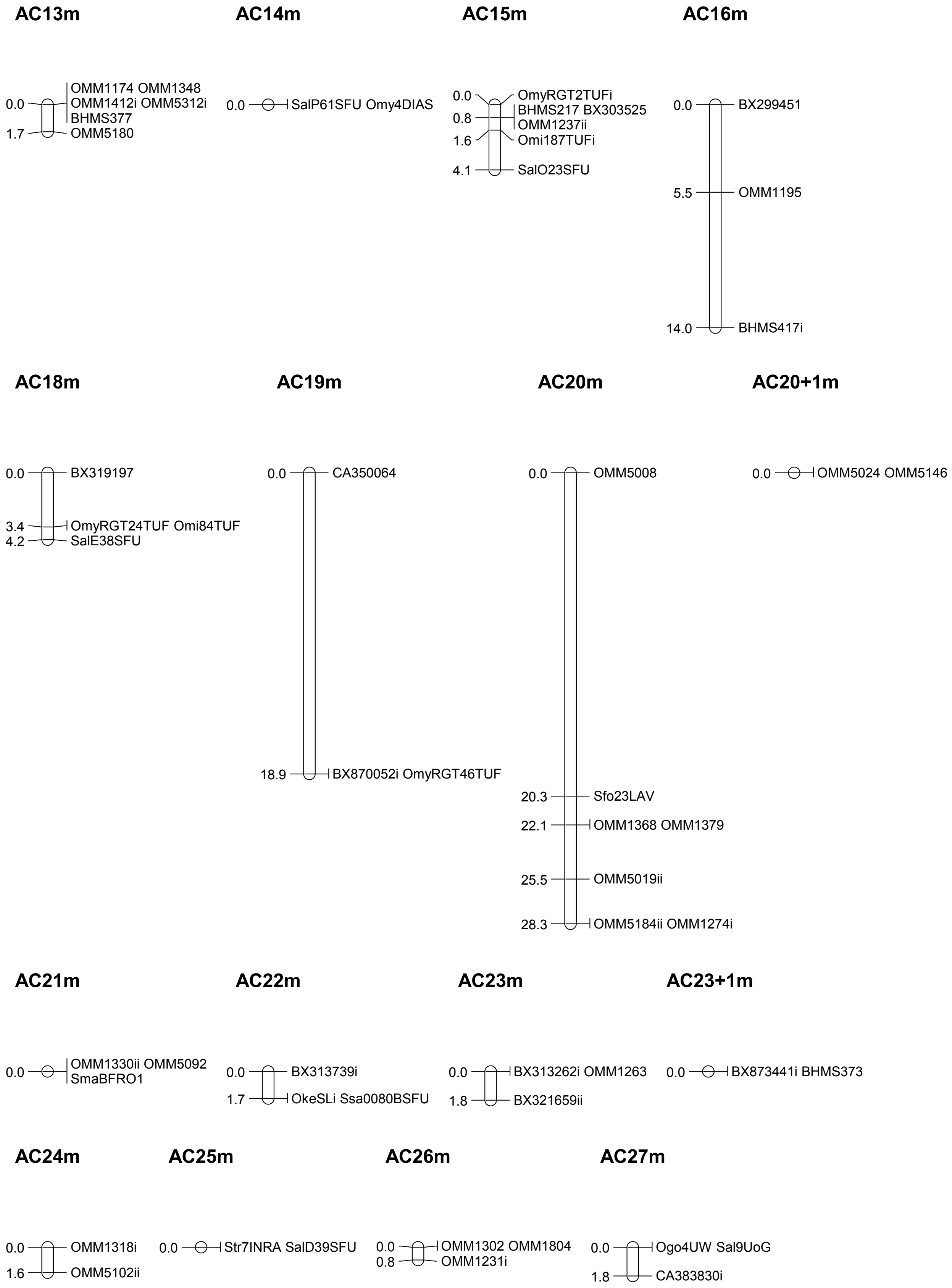
AC13m
0.0 OMM1174 OMM1348 OMM1412i OMM5312i BHMS377
1.7 OMM5180
AC14m
0.0 SalP61SFU Omy4DIAS
AC15m
0.0 OmyRGT2TUFi
0.8 BHMS217 BX303525 OMM1237ii
1.6 Omi187TUFi
4.1 SalO23SFU
AC16m
0.0 BX299451
5.5 OMM1195
14.0 BHMS417i
AC18m
0.0 BX319197
3.4 OmyRGT24TUF Omi84TUF
4.2 SalE38SFU
AC19m
0.0 CA350064
18.9 BX870052i OmyRGT46TUF
AC20m
0.0 OMM5008
20.3 Sfo23LAV
22.1 OMM1368 OMM1379
25.5 OMM5019ii
28.3 OMM5184ii OMM1274i
AC20+1m
0.0 OMM5024 OMM5146
AC21m
0.0 OMM1330ii OMM5092 SmaBFRO1
AC22m
0.0 BX313739i
1.7 OkeSLi Ssa0080BSFU
AC23m
0.0 BX313262i OMM1263
1.8 BX321659ii
AC23+1m
0.0 BX873441i BHMS373
AC24m
0.0 OMM1318i
1.6 OMM5102ii
AC25m
0.0 Str7INRA SalD39SFU
AC26m
0.0 OMM1302 OMM1804
0.8 OMM1231i
AC27m
0.0 Ogo4UW Sal9UoG
1.8 CA383830i

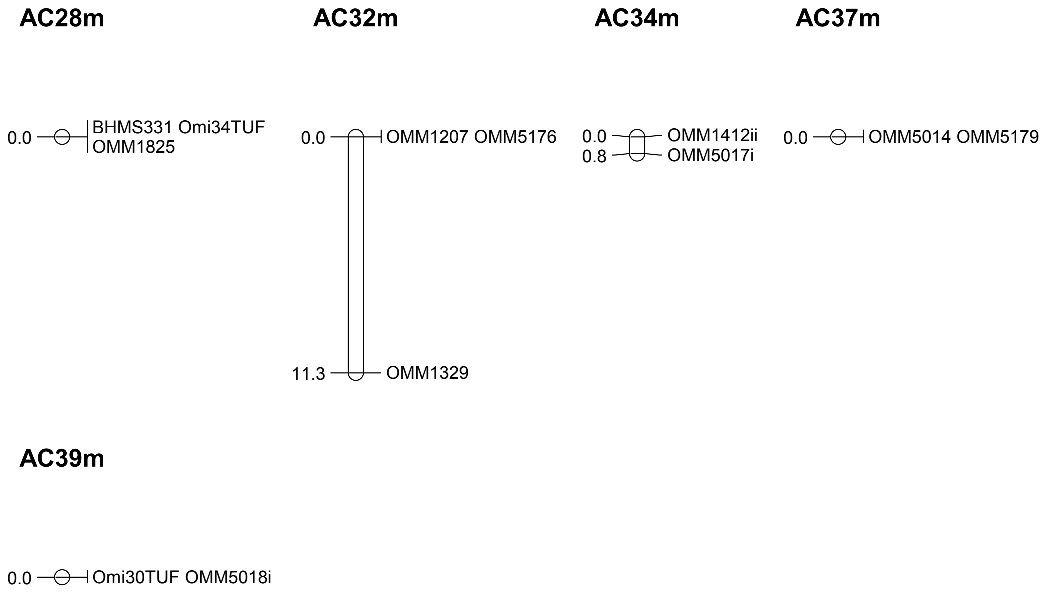
AC28m
0.0
BHMS331 Omi34TUF
OMM1825
AC32m
0.0
OMM1207 OMM5176
11.3
OMM1329
AC34m
0.0
OMM1412ii
0.8
OMM5017i
AC37m
0.0
OMM5014 OMM5179
AC39m
0.0
Omi30TUF OMM5018i
