# Supplementary material for: The genetic basis of salinity tolerance traits in Arctic charr (Salvelinus alpinus)
Source: BMC Genet. 2011 Sep 21;12:81. doi: 10.1186/1471-2156-12-81 (PMC3190344; doi:10.1186/1471-2156-12-81)
Supplement: Additional file 5 — QTL for Na+/K+-ATPase activity and blood plasma osmolality in two Arctic charr (Salvelinus alpinus) full-sib families. [file 1471-2156-12-81-S5.PDF]

Additional File 5 - QTL for Na^+/K^+-ATPase activity and blood plasma osmolality in two Arctic charr (*Salvelinus alpinus*) full-sib families.

LG linkage group; PEV proportion of experimental variation. All QTL were detected using interval analysis.

LG/Trait	Parent[1]/Family	Marker/Interval	*P*-value	PEV
**Na^+/K^+-ATPase activity**				
1	F/10	OMM1330i	0.055	0.051
1	F/12	CB512520 - CA379795	0.031	0.092
4	F/10	BX866899	0.025	0.066
5	F/10	Omy1339INRA - OMM5295	0.041	0.081
5	M/10	BX076085 - Ots517NWFSC	0.045	0.068
7	F/10	BX309199 - Omy10INRA	0.030	0.087
11	F/12	Ssa0054BSFU - BHMS7.011	0.028	0.072
15	F/10	OmyRGT2TUFi - BX303525	0.047	0.077
17	F/12	OMM5287 - OMM5133	0.030	0.060
18	M/12	BX319197 - OmyRGT24TUF	0.034	0.076
25	M/12	Str7INRA - SalD39SFU	0.008	0.089
28	M/12	OMM1459	0.021	0.082
32	M/12	OMM5176 - OMM1329	0.032	0.087
**Blood plasma osmolality**				
4	F/10	Ots500NWFSC - Ssa32OSL	0.029	0.080
4	M/12	Omy6DIAS - OMM5137	0.009	0.141
12	F/12	BX879524ii - CA383830i	0.001	0.131
20	F/10	OMM5024, OMM5146	0.031	0.199
20	M/12	OMM5008	0.049	0.093
27	F/12	CA345149	0.026	0.092
31	M/10	OMM1290	0.041	0.074
32	M/12	OMM5176 - OMM1329	0.064	0.93

[1] F denotes female while M denotes male.
