# Supplementary material for: The genetic basis of salinity tolerance traits in Arctic charr (Salvelinus alpinus)
Source: BMC Genet. 2011 Sep 21;12:81. doi: 10.1186/1471-2156-12-81 (PMC3190344; doi:10.1186/1471-2156-12-81)
Supplement: Additional file 6 — QTL for growth in sea water in two Arctic charr (Salvelinus alpinus) full-sib families. [file 1471-2156-12-81-S6.PDF]

**Additional file 6 - QTL for growth in sea water in two Arctic charr (*Salvelinus alpinus*) full-sib families.**

LG linkage group; PEV proportion of experimental variation. All QTL were detected using interval analysis.

| LG/Trait | Parent[1]/Family | Marker/Interval | *P*-value | PEV |
|---|---|---|---|---|
| **Specific growth rate 1 (June 12 to August 28, 2008)** | | | | |
| 1 | F/12 | CB512520 - CA379795 | 0.032 | 0.067 |
| 4 | F/10 | OMM5175 | 0.025 | 0.048 |
| 5 | M/12 | OMM5295 | 0.023 | 0.053 |
| 7 | F/12 | BX309199 - Omy10INRA | 0.039 | 0.076 |
| 18 | F/12 | OmyRGT24TUF - Omi84TUF | 0.011 | 0.068 |
| 19 | F/12 | BX870052i - OmyRGT46TUF | 0.042 | 0.068 |
| 19 | M/12 | CA350064 - OmyRGT46TUF | 0.003 | 0.142 |
| 20 | F/12 | BX318599i | 0.016 | 0.058 |
| 22 | F/12 | BX313739i - Ssa0080BSFU | 0.002 | 0.103 |
| 26 | M/12 | OMM1302 - OMM1231i | 0.043 | 0.041 |
| 34 | F/10 | OMM1657i - OMM5312i | 0.024 | 0.062 |
| **Specific growth rate 2 (August 29 to November 14, 2008)** | | | | |
| 1 | F/10 | CB512520 - Omi60TUF | 0.033 | 0.105 |
| 4 | F/10 | BX866899 | 0.042 | 0.047 |
| 7 | M/12 | BX309199 - BHMS7.036 | 0.029 | 0.05 |
| 8 | F/10 | BG934221 - BX305863 | 0.018 | 0.085 |
| 8 | M/10 | BX305863 - OMM5061 | 0.037 | 0.066 |
| 13 | M/12 | OMM1174 - OMM5180 | 0.004 | 0.085 |
| 14 | M/12 | SalP61SFU - Omy4DIAS | 0.014 | 0.071 |
| 16 | M/12 | BX299451 - OMM1195 | 0.04 | 0.075 |
| 18 | F/12 | OmyRGT24TUF - Omi84TUF | 0.015 | 0.072 |
| 20 | F/10 | OMM5184 | 0.027 | 0.063 |
| 21 | F/12 | OMM1330ii - OMM5074ii | 0.002 | 0.118 |
| 22 | F/12 | BX313739i - Ssa0080BSFU | 0.035 | 0.055 |
| 23 | M/12 | OMM1263 - BX321659ii | 0.045 | 0.045 |
| 26 | M/12 | OMM1804 - OMM1231i | 0.035 | 0.049 |
| 37 | M/12 | BX310634 | 0.039 | 0.051 |

[1] F denotes female while M denotes male.
