# Supplementary material for: The genetic basis of salinity tolerance traits in Arctic charr (Salvelinus alpinus)
Source: BMC Genet. 2011 Sep 21;12:81. doi: 10.1186/1471-2156-12-81 (PMC3190344; doi:10.1186/1471-2156-12-81)
Supplement: Additional file 7 — QTL for salinity tolerance traits based on a combined analysis of two Arctic charr (Salvelinus alpinus) full-sib families. [file 1471-2156-12-81-S7.PDF]

Additional file 7 - QTL for salinity tolerance traits based on a combined single-marker analysis of two Arctic charr (*Salvelinus alpinus*) full-sib families.

LG linkage group; PEV proportion of experimental variation.

LG/Trait	Marker	Parent[1]/Family	*P*-value[2]	PEV
**Na^+/K^+-ATPase activity**				
7	Omy10INRA	F/10	0.041	0.087
		M/10		0.00014
		F/12		0.003
		M/12		0.049
15	OmyRGT2TUFi	F/10	0.053	0.085
		M/10		N/A
		F/12		N/A
		M/12		N/A
**Blood plasma osmolality**				
4	Ssa32OSLi	F/10	0.031	0.067
		M/10		0.005
		F/12		0.058
		M/12		0.177
20	OMM5008	F/10	0.056	0.063
		M/10		0.063
		F/12		0.023
		M/12		0.077
**Specific growth rate 1 (June 12 to August 28, 2008)**				
7	BX073974	F/10	0.048	0.032
		M/10		0.018
		F/12		0.052
		M/12		0.011
19	BX870052i	F/10	0.001[2]	0.0073
		M/10		0.0096
		F/12		0.049
		M/12		0.105
22	OkeSLi	F/10	0.042	0.0015
		M/10		0.002
		F/12		0.08
		M/12		0.011
26	OMM1302	F/10	0.024	0.07
		M/10		0.0043
		F/12		0.03
		M/12		0.036
**Specific growth rate 2 (August 29 to November 14, 2008)**				
8	OmyRGT6TUFi	F/10	0.003[2]	0.075
		M/10		0.05
		F/12		0.032
		M/12		0.0007
14	SalP61SFU, BHMS238, Omy4DIAS	F/10	0.005[2]	0.0021
		M/10		0.0038

LG/Trait	Marker	Parent[1]/Family	*P*-value[2]	PEV
		F/12		0.029
		M/12		0.061
15	OMM1237ii	F/10	0.043	0.028
		M/10		0.022
		F/12		0.017
		M/12		0.0012
22	BX313739i	F/10	0.018	0.0034
		M/10		0.041
		F/12		0.046
		M/12		0.00061

[1] F denotes female while M denotes male.

[2] QTL with genome-wide significance.
