# Supplementary material for: The genetic basis of salinity tolerance traits in Arctic charr (Salvelinus alpinus)
Source: BMC Genet. 2011 Sep 21;12:81. doi: 10.1186/1471-2156-12-81 (PMC3190344; doi:10.1186/1471-2156-12-81)
Supplement: Additional file 8 — QTL homeologies for seawater and body size traits in Arctic charr (Salvelinus alpinus). [file 1471-2156-12-81-S8.PDF]

Additional File 8 - QTL homeologies for salinity tolerance and body size traits in Arctic charr

	1	3	4	5	7	8	9	11	12	13	14	16	17	18	19	20	21	24	25	26	27	28	32	34
**1**	1		N,S1, S2					N								S1, S2	S2							
**3**		3																						
**4**	B3, K3		4		N, S1, S2										S1				N			N		
**5**				5															N					
**7**			B3		7								N						N					
**8**						8								S2			S2							
**9**			B3, K3				9																	
**11**	B1, K3							11																
**12**									12												O			
**13**	B1, K2, K3									13	S2	S2												
**14**										K2	14													
**16**										K3		16												
**17**					B1								17											
**18**														18					N					
**19**			B3												19									
**20**	K1, K3															20								
**21**	B2, K1, K2, K3					K3		K3									21							
**24**		B3, K1, K2				K3												24			SW QTL Homeologies			
**25**			B3, K3		B1, B2, B3									B3, K1					25					
**26**																				26				
**27**									B1												27			
**28**			K3															BW and K QTL Homeologies				28		
**32**															B1, B2								32	
**34**										B1														34

Syntenic block with ≥ 2 markers supporting a homeology

Homeology supported by a single marker

Homeologous linkage groups where putative homeologous QTL were not detected

N Na+/K+-ATPase activity (NKAA)

O Blood plasma osmolality (OSMO)

S1 Specific growth rate 1 (SGR1)

S2 Specific growth rate 2 (SGR2)

B1 Body weight 1 (BW1)

B2 Body weight 2 (BW2)

B3 Body weight 3 (BW3)

K1 Condition factor 1 (K1)

K2 Condition factor 2 (K2)

K3 Condition factor 3 (K3)

**** Homeologous relationships and synteny information obtained from Timusk et al. 2011.***
