# Supplementary material for: The genetic basis of salinity tolerance traits in Arctic charr (Salvelinus alpinus)
Source: BMC Genet. 2011 Sep 21;12:81. doi: 10.1186/1471-2156-12-81 (PMC3190344; doi:10.1186/1471-2156-12-81)
Supplement: Additional file 9 — QTL for body weight and Fulton's condition factor in two Arctic charr (Salvelinus alpinus) full-sib families. [file 1471-2156-12-81-S9.PDF]

Additional file 9 - QTL for body weight and Fulton's condition factor in two Arctic charr (*Salvelinus alpinus*) full-sib families.

LG linkage group; PEV proportion of experimental variation. All QTL were detected using interval analysis.

LG/Trait	Parent[1]/Family	Marker/Interval	*P*-value	PEV
**Body weight on June 12, 2008**				
1	M/10	OMM1300 - Ssa77NUIG	0.051	0.041
1	M/12	Omy21INRAii - OMM5074ii	0.016	0.063
7	F/10	Omy10INRA	0.016	0.065
10	M/12	OMM1237i - Omi187TUFii	0.044	0.038
11	M/10	Ssa0054BSFU - OmyRGT35TUFi	0.022	0.056
12	F/10	Sal9UoG - CA383830ii	0.047	0.046
13	M/10	OMM1211 - OMM1412ii	0.04	0.039
17	F/12	OMM5287 - OMM5133	0.051	0.034
19	M/10	BX870052i - OmyRGT46TUF	0.011	0.076
24	M/12	OMM1318i - OMM5102ii	0.009	0.066
25	F/10	SalD39SFU	0.01	0.06
26	F/10	OMM1804 - OMM1231i	0.05	0.051
27	M/10	CA383830i - Sal9UoG	0.005	0.101
28	M/12	OMM1459	0.006	0.06
32	F/12	OMM5176 - BHMS7.020	0.001	0.129
34	F/10	OMM1657i - OMM1412i	0.047	0.055
**Body weight on August 28, 2008**				
1	M/12	Omy21INRAii - OMM5074ii	0.035	0.051
7	F/10	BX309199 - Omy10INRA	0.021	0.061
19	F/12	BX870052i	0.01	0.08
21	F/12	OMM1330ii - OMM5074ii	0.027	0.068
24	F/10	OMM1318i - OMM5102ii	0.047	0.044
24	M/12	OMM1318i - OMM5102ii	0.014	0.059
25	F/10	SalD39SFU	0.034	0.043
26	F/12	OMM1804	0.048	0.04
27	M/10	CA383830i - Sal9UoG	< 0.001	0.172
32	F/12	OMM5176 - BHMS7.020	0.004	0.098
37	M/10	OMM1270 - BX310634	0.026	0.046
43	F/10	Sfo23LAV - OMM1379	0.034	0.043
**Body weight on November 14, 2008**				
1	F/10	OMM1300	0.033	0.046
3	M/10	OMM1318i - BHMS161	0.021	0.072
4	F/10	BX866899	0.041	0.039
7	F/10	BX309199 - Omy10INRA	0.001	0.106
9	F/12	BX881655i - TC126859	0.032	0.05
12	F/10	Sal9UoG - CA383830ii	0.021	0.067
12	M/10	OMM1345i - BX879524ii	0.029	0.054
18	F/12	OmyRGT24TUF - Omi84TUF	0.043	0.039
18	M/10	SalE38SFU - OMM1442i	0.047	0.046

LG/Trait	Parent[1]/Family	Marker/Interval	*P*-value	PEV
24	F/10	OMM1318i - OMM5102ii	0.05	0.051
25	M/12	Str7INRA - SalD39SFU	0.043	0.042
26	F/12	OMM1804	0.037	0.042
32	F/12	OMM5176 - BHMS7.020	0.022	0.076
37	M/12	BX310634	0.045	0.037
**Condition factor on June 12, 2008**				
1	F/10	CB512520 - Omi60TUF	0.018	0.098
1	F/12	CB512520	0.049	0.039
1	M/10	OMM1330i - OMM1300	< 0.001	0.106
1	M/12	OMM5074i - BX311884i	0.009	0.072
3	M/10	OMM1318i - BHMS161	0.047	0.054
10	F/10	CA054565	0.046	0.039
17	F/10	OMM5133 - BHMS7.036i	0.028	0.049
18	F/12	BX079862i - SalE38SFU	0.037	0.045
20	F/10	BX890355i - OMM5184i	0.032	0.065
20	M/10	BX890355i - OMM5184i	0.04	0.065
21	M/10	Omi20TUF	0.039	0.085
23	M/10	OMM1372ii - BX873441i	0.032	0.038
24	M/12	OMM1318i - OMM5102ii	0.009	0.06
26	M/12	OMM1804 - OMM1231i	0.017	0.049
28	F/12	Omi34TUF - OMM1825	0.002	0.126
28	M/12	BHMS331 - Omi34TUF	0.035	0.034
31	F/10	OMM1290	0.001	0.086
36	F/12	CA061336 - CA359625	0.027	0.04
37	F/12	OMM6179 - BX310634	0.022	0.047
**Condition factor on August 28, 2008**				
1	M/10	OMM1330i - OMM1300	0.034	0.042
3	M/10	Str11INRA - Ots101SSBI	0.002	0.095
13	F/10	OMM1211	0.011	0.059
14	F/12	SalP61SFU - BHMS238	0.031	0.043
18	F/10	BX319197	0.044	0.038
21	M/10	Omi20TUF	0.012	0.115
22	F/10	BX313739i - OkeSLi	0.029	0.039
24	F/10	OMM1318i - OMM5102ii	0.002	0.088
27	M/10	CA383830i - Sal9UoG	0.019	0.069
28	M/10	Omi34TUF - OMM1825	0.001	0.083
39	M/10	OMM5236 - OMM5289	0.012	0.06
**Condition factor on November 14, 2008**				
1	M/12	BX311884i - OMM1300	0.015	0.072
4	F/10	BX866899	0.025	0.043
8	F/10	OmyRGT6TUFi - BX305863	0.032	0.069
8	F/12	BG934221 - BX305863	0.025	0.083
9	F/10	Ssa0072BSFU - TC126859	0.027	0.051
11	F/12	Ssa0054BSFU - BHMS7.011	0.038	0.042
13	F/10	OMM1211	0.004	0.083
13	M/10	OMM1211 - OMM1412ii	0.033	0.051

LG/Trait	Parent[1]/Family	Marker/Interval	*P*-value	PEV
15	F/10	OmyRGT2TUFi - BX303525	0.033	0.056
15	M/10	BHMS217	0.046	0.039
16	F/10	OMM1195 - BHMS417i	0.038	0.043
16	M/12	BX299451 - OMM1195	0.023	0.073
20	M/12	OMM5019ii - OMM5184ii	0.051	0.054
21	F/12	CA344270	0.026	0.044
21	M/12	OMM5092 - SmaBFRO1	0.038	0.044
24	F/10	OMM1318i - OMM5102ii	0.01	0.073
25	M/10	Str7INRA - SalD39SFU	0.01	0.066
28	M/10	OMM1307 - OMM1459	0.023	0.054
37	M/12	BX310634	0.04	0.043

[1] F denotes female while M denotes male.
