# Supplementary material for: The genetic basis of salinity tolerance traits in Arctic charr (Salvelinus alpinus)
Source: BMC Genet. 2011 Sep 21;12:81. doi: 10.1186/1471-2156-12-81 (PMC3190344; doi:10.1186/1471-2156-12-81)
Supplement: Additional file 10 — QTL for body weight and condition factor based on a combined analysis of two Arctic charr (Salvelinus alpinus) full-sib families. [file 1471-2156-12-81-S10.PDF]

Additional file 10 - QTL for body weight and condition factor based on a combined single-marker analysis of two Arctic charr (*Salvelinus alpinus*) full-sib families.

LG linkage group; PEV proportion of experimental variation.

LG/Trait	Marker	Parent[1]/Family	*P*-value[2]	PEV
**Body weight on June 12, 2008**				
1	CB512520	F/10	0.022[2]	0.027
		M/10		0.041
		F/12		0.015
		M/12		0.062
19	OmyRGT46TUF	F/10	0.006[2]	0.0046
		M/10		0.076
		F/12		0.027
		M/12		0.018
**Body weight on August 28, 2008**				
19	BX870052i	F/10	0.012[2]	0.0043
		M/10		0.042
		F/12		0.08
		M/12		0.0081
**Body weight on November 14, 2008**				
3	OMM1318i	F/10	0.037	0.019
		M/10		0.072
		F/12		0.0098
		M/12		0.01
7	Omy10INRA	F/10	0.039	0.099
		M/10		0.0028
		F/12		0.0018
		M/12		0.0069
19	OmyRGT46TUF	F/10	0.047	0.0063
		M/10		0.044
		F/12		0.029
		M/12		0.028
**Condition factor on June 12, 2008**				
1	CB512520	F/10	0.001	0.098
		M/10		0.097
		F/12		0.018
		M/12		0.061
28	Omi34TUF	F/10	0.014[2]	0.0043
		M/10		0.021
		F/12		0.108
		M/12		0.034
**Condition factor on August 28, 2008**				
3	Ots101SSBI, Str11INRA	F/10	0.034	0.0013
		M/10		0.095
		F/12		0.0034

LG/Trait	Marker	Parent[1]/Family	*P*-value[2]	PEV
		M/12		0.0053
14	SalP61SFU, BHMS238, Omy4DIAS	F/10	0.028	0.028
		M/10		0.012
		F/12		0.043
		M/12		0.0064
26	OMM1231i	F/10	0.038	
		M/10		
		F/12		
		M/12		
28	Omi34TUF	F/10	0.014[2]	0.00034
		M/10		0.083
		F/12		0.022
		M/12		0.022
**Condition factor on November 14, 2008**				
8	OMM1429	F/10	0.04	0.069
		M/10		0.00012
		F/12		0.058
		M/12		0.0053
9	Ssa0072BSFU	F/10	0.044	0.051
		M/10		0.034
		F/12		0.016
		M/12		0.0029
16	BX299451	F/10	0.026	0.04
		M/10		0.00012
		F/12		0.0063
		M/12		0.073
20	OMM5019i	F/10	0.012[2]	0.055
		M/10		0.055
		F/12		0.048
		M/12		0.065

[1] F denotes female while M denotes male.

[2] QTL with genome-wide significance.
